# Cross-Kingdom Comparative Transcriptomics Reveals Conserved Genetic Modules in Response to Cadmium Stress

**DOI:** 10.1128/mSystems.01189-21

**Published:** 2021-12-07

**Authors:** Miaomiao Chen, Likun Wang, Xin Zheng, Michael Cohen, Xiaofang Li

**Affiliations:** a Hebei Key Laboratory of Soil Ecology, Centre for Agricultural Resources Research, Institute of Genetics and Developmental Biology, Chinese Academy of Sciences, Shijiazhuang, China; b University of Chinese Academy of Sciences, Beijing, China; c Sonoma State University, Rohnert Park, California, USA; Pacific Northwest National Laboratory

**Keywords:** cross-kingdom, comparative transcriptomics, cadmium resistance, *Chlamydomonas reinhardtii*, *Escherichia coli*, *Saccharomyces cerevisiae*

## Abstract

It is known that organisms have developed various mechanisms to cope with cadmium (Cd) stress, while we still lack a system-level understanding of the functional isomorphy among them. In the present study, a cross-kingdom comparison was conducted among Escherichia coli, Saccharomyces cerevisiae, and Chlamydomonas reinhardtii, through toxicological tests, comparative transcriptomics, as well as conventional functional genomics. An equivalent level of Cd stress was determined via inhibition tests. Through transcriptome comparison, the three organisms exhibited differential gene expression under the same Cd stress relative to the corresponding no-treatment control. Results from functional enrichment analysis of differentially expressed genes (DEGs) showed that four metabolic pathways responsible for combating Cd stress were commonly regulated in the three organisms, including antioxidant reactions, sulfur metabolism, cell wall remodeling, and metal transport. *In vivo* expression patterns of 43 DEGs from the four pathways were further examined using quantitative PCR and resulted in a relatively comparable dynamic of gene expression patterns with transcriptome sequencing (RNA-seq). Cross-kingdom comparison of typical Cd stress-responding proteins resulted in the detection of 12 groups of homologous proteins in the three species. A class of potential metal transporters were subjected to cross-transformation to test their functional complementation. An ABC transporter gene in E. coli, possibly homologous to the yeast *ycf1*, was heterologously expressed in S. cerevisiae, resulting in enhanced Cd tolerance. Overall, our findings indicated that conserved genetic modules against Cd toxicity were commonly regulated among distantly related microbial species, which will be helpful for utilizing them in modifying microbial traits for bioremediation.

**IMPORTANCE** Research is establishing a systems biology view of biological response to Cd stress. It is meaningful to explore whether there is regulatory isomorphy among distantly related organisms. A transcriptomic comparison was done among model microbes, leading to the identification of a conserved cellular model pinpointing the generic strategies utilized by microbes for combating Cd stress. A novel E. coli transporter gene substantially increased yeast’s Cd tolerance. Knowledge on systems understanding of the cellular response to metals provides the basis for developing bioengineering remediation technology.

## INTRODUCTION

Soil heavy metal pollution poses a significant threat to the quality of farmland soil and underground water, as well as food safety and human health. The exploration of metal resistance mechanisms has attracted unprecedented attention in recent years owing to their importance in bioremediation practices (1, 2). Numerous studies have detected heavy metal-resistant mechanisms of specific organisms with remediation potentials, and what is more, modifying them using bioengineering technology (3, 4). However, a systemic understanding of metal stress responses across distantly related organisms is also of great interest, as these shared traits may be more easily applied in bioengineering strains, and can finally be used in a wider range in bioremediation.

Cadmium (Cd) is one of the most ubiquitous metallic pollutants worldwide. System-level exploration of cellular responses to Cd stress has been reported for both prokaryotic and eukaryotic organisms, while most of the exploration of cellular responses was done for specific species (5–8). For example, after exposure to Cd, genes encoding proteins involved in the synthesis of phenols and metallothioneins in the fungus Paxillus involutus were upregulated, resulting in the incremental production of cysteine-enriched compounds (9). Pleurotus ostreatus responded to Cd stress by modulating cell processes, including cell wall remodeling, Cys-enriched compound synthesis, reactive oxygen species (ROS) response, and metal transport in a systematic manner (5). Likewise, a comparative transcriptomic study showed that a low-Cd wheat variety mobilized a wide spectrum of cellular activities such as enriched ion binding, antioxidant defense mechanisms, sulfotransferase activity, and cysteine (Cys) biosynthetic process to reduce Cd concentration (10). Existing research has deepened our understanding on systems-level Cd responses in specific organisms, while it is also critical to explore the conserved mechanisms that have been selected by nature during evolution, as these conserved modules may play an important role in facilitating stress tolerance (11).

Comparative genomics or transcriptomic analysis allows us to search for conserved and specific genetic elements among different species (12, 13), especially for comparing among distantly related species (14, 15). Several heavy metal-responding mechanisms have been identified through transcriptomic comparisons (5–8). For instance, based on a comparative transcriptomic study, Song et al. (6) found that activation of redox homeostasis and oxidation-related metabolic processes were the primary response to Cd stress in switchgrass roots, and the *hsp* (heat shock protein) gene was able to improve plant tolerance against Cd significantly. Between- and within-species comparisons of transcriptomic profiles of *Arabidopsis* and Thlaspi caerulescens showed that lignin, glutathione (GSH), and sulfate metabolism were involved in Cd accumulation (16). A comparative transcriptomic analysis between two pak choi (Brassica rapa L. subsp. *chinensis*) cultivars demonstrated that plasma membrane and tonoplast-localized transport genes were related to Cd accumulation (17). Comparative analysis of high-Cd-accumulating Solanum nigrum and low-Cd-accumulating Solanum torvum indicated that Fe deficiency transporters might play a role in the differential uptake of Cd (18).

Due to tremendous differences in gene expression and the unevenness of annotation information among cross-kingdom organisms that can be referenced, only a limited number of studies targeted comparing distantly related species. Ferrari et al. found that genes related to biological rhythm were conservatively expressed by comparing the diurnal transcriptional programs of nine members of *Archaeplastida*, including eukaryotic algae, terrestrial plants, and *Cyanobacteria* (14). Coexpression modules were found to be shared by three distantly related metazoan phyla, human, worm, and fly, revealing ancient and conserved features in animal development (19). Moreover, a cross-phylum comparative transcriptomic analysis of 10 species discovered conserved early and late phases of development in vertebrates (20). These studies intimated the existence of a universal gene regulation mode and indicated the feature of older phylostrata genes presenting stronger conservation (14). To date, conserved genetic modules for Cd stress response among distantly related species have not been well studied.

Escherichia coli, Saccharomyces cerevisiae, and Chlamydomonas reinhardtii are commonly used as model organisms for metal resistance research in the kingdoms of bacteria, fungi, and protozoa (algae), respectively (21–25). Some specific Cd response pathways and genes of these three organisms have been clearly described in previous studies. In E. coli, Cd efflux genes like *znt*, *czc*, and *cad* systems were found to be responsible for Cd detoxification (24, 26–28). Biosynthesis of GSH, antioxidant enzymes, and transporters were induced in S. cerevisiae to cope with Cd stress (29, 30). C. reinhardtii is able to mobilize photosystem remodeling, chlorophyll biosynthesis, *S*-adenosylmethionine (SAM) synthesis, iron uptake, and phytochelatin-Cd (PC-Cd) complex sequestration to alleviate Cd toxicity (31–33). For biological Cd stress response, shared Cd-responding genetic modules cannot be inferred from existing studies due to the lack of a uniform experimental regime.

This study was undertaken to assess the gene expression variation in E. coli, S. cerevisiae, and C. reinhardtii under equivalent levels of Cd stress and the conserved genetic modules among them using a comparative transcriptomic method. The selective common modules were cross-transformed among the three organisms to verify their functional complementation in Cd resistance. Findings from this study may advance a systems biology understanding of microbial Cd responses, and the detected shared pathways among the tested species can be utilized in modifying microbial traits for bioremediation.

## RESULTS

### Phenotypic response of the tested species to Cd stress.

**FIG 1:**
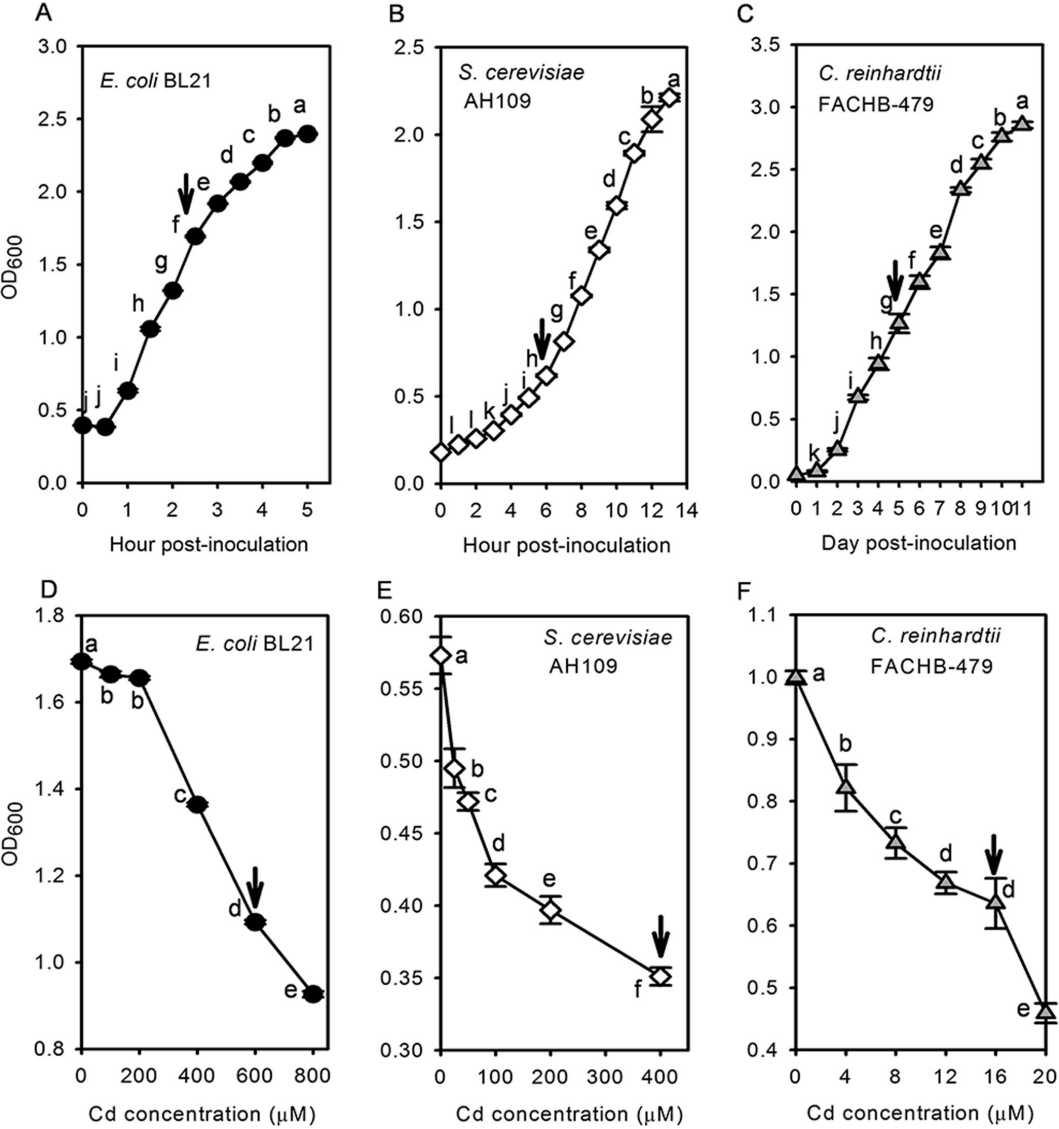
Growth patterns of the three tested species under Cd stress. (A to C) Growth curves of Escherichia coli BL21, Saccharomyces cerevisiae AH109, and Chlamydomonas reinhardtii FACHB-479, respectively, for determination of the logarithmic growth phase. (D to F) Dose-response curves using cell density as an indicator of the tested species (E. coli BL21, S. cerevisiae AH109, and C. reinhardtii FACHB-479) against a specific Cd concentration gradient. Arrows represent the points selected for subsequent transcriptome experiments. Bars with different letters are significantly different at *P* ≤ 0.05 (one-way ANNOVA).

Logarithmic growth phase of the three strains was determined by monitoring their growth rates. E. coli BL21 presented rapid growth in the first 3 h postinoculation; therefore, 2.5 h postinoculation was selected as the sampling time point (Fig. 1A). After 2.5 h of 100 μM, 200 μM, 400 μM, 600 μM, or 800 μM Cd treatment in liquid LB medium, the growth inhibition rate was 2.0%, 2.2%, 19.5%, 35.5%, and 45.3%, respectively. As a result, cells of E. coli BL21 treated by being cultured with 600 μM Cd for 2.5 h were used for subsequent transcriptome sequencing (Fig. 1D). Growth of S. cerevisiae AH109 reached logarithmic phase 6 h after inoculation, and 6 h was selected as the Cd treatment time (Fig. 1B). Growth of S. cerevisiae AH109 was inhibited by 13.6%, 17.6%, 26.5%, 30.7%, and 38.7% upon exposure to 25 μM, 50 μM, 100 μM, 200 μM, and 400 μM Cd, respectively. Thus, 400 μM Cd was used as Cd treatment concentration for S. cerevisiae AH109 (Fig. 1E). C. reinhardtii FACHB-479 presented a higher sensitivity to Cd than E. coli BL21 and S. cerevisiae AH109. The C. reinhardtii FACHB-479 strain started logarithmic growth phase 1 h postinoculation. After 4 μM, 8 μM, 12 μM, 16 μM, or 20 μM Cd treatment, the growth of C. reinhardtii FACHB-479 was inhibited by 17.8%, 26.7%, 33.1%, 36.4%, and 54.1%, respectively. Thus, 12 μM Cd treatment was selected for C. reinhardtii FACHB-479 (Fig. 1C and F).

### Transcriptome sequencing and annotation.

Approximately 104.60 million of 150-bp paired-end reads were generated for E. coli BL21, 363.09 million reads for S. cerevisiae AH109, and 403.76 million reads for C. reinhardtii FACHB-479. After quality trimming, the Q30 of the retained high-quality reads of E. coli BL21, S. cerevisiae AH109, and C. reinhardtii FACHB-479 were 95.06, 96.38, and 94.46, respectively (see Table S1 in the supplemental material). Data from each sample were subsequently merged and mapped to the reference genome sequences, resulting in overall 99%, 96% and 95% total mapped reads. Among the mapped reads, 98.40%, 91.54%, and 95.39% of these reads were uniquely mapped, including 4,141, 5,774, and 14,435 identified unigenes for E. coli BL21, S. cerevisiae AH109, and C. reinhardtii FACHB-479, respectively (see Fig. S1 in the supplemental material). Using the cutoff of the absolute value of log_2_ fold change (FC) of ≥1 and adjusted *P* value (*p* adjust) of <0.05, a total of 541, 80, and 5126 DEGs were detected in E. coli BL21, S. cerevisiae AH109, and C. reinhardtii FACHB-479, respectively (Fig. S1). The genes were considered upregulated if the transcript level in Cd-treated cells was higher than the level in the control cells; conversely, genes were considered downregulated if the transcript level in the Cd treatment was lower than the value for the control cells.

10.1128/mSystems.01189-21.1 FIG S1 Number of genes and number of differentially expressed genes (DEGs) identified in Escherichia coli BL21, Saccharomyces cerevisiae AH109, and Chlamydomonas reinhardtii FACHB-479. (A to C) Number of unigenes identified in E. coli BL21, S. cerevisiae AH109, and C. reinhardtii FACHB-479, respectively, and visualized in Venn diagrams. (D to F) Number of identified DEGs in E. coli BL21, S. cerevisiae AH109, and C. reinhardtii FACHB-479, respectively, and visualized in volcano plots. DEGs were considered the absolute value of log_2_ FC ≥ 1 and *p* adjust < 0.05. Download FIG S1, TIF file, 5.5 MB. Copyright © 2021 Chen et al. 2021 Chen et al. https://creativecommons.org/licenses/by/4.0/ This content is distributed under the terms of the Creative Commons Attribution 4.0 International license.

10.1128/mSystems.01189-21.4 TABLE S1 Statistics of RNA-seq read mapping of E. coli BL21, S. cerevisiae AH109, and C. reinhardtii FACHB-479^z^. Download Table S1, DOCX file, 0.01 MB. Copyright © 2021 Chen et al. 2021 Chen et al. https://creativecommons.org/licenses/by/4.0/ This content is distributed under the terms of the Creative Commons Attribution 4.0 International license.

Gene sequences of the three organisms were searched against the Gene Ontology (GO), Kyoto Encyclopedia of Genes and Genomes (KEGG), NR, Swiss-Prot, and COG databases, resulting in 3,559 (82.02%), 2,764 (63.7%), 4,321 (99.59%), 4,028 (92.83%), and 4,001 (92.21%) annotated unigenes found in E. coli BL21, respectively; 6,685 (93.81%), 3,643 (51.12%), 2,281 (32.00%), 4,987 (69.98%), and 2,706 (37.97%) annotated unigenes in S. cerevisiae AH109, respectively; and 8,637 (48.14%), 6,337 (35.32%), 11,190 (62.36%), 17,662 (98.43%), and 8,001 (44.61%) annotated unigenes, respectively, in C. reinhardtii FACHB-479.

### Functional enrichment analysis.

GO enrichment analysis was performed for all the DEGs in the three organisms, and GO terms that commonly enriched in all the three organisms were selected (Fig. S2). It turned out that GO related to ion transport, protein metabolism, and organelles were significantly enriched in the three organisms (Fig. S2). For further functional analysis, significantly responsive genes were selected at the threshold of the absolute value of log_2_ FC of ≥1 and *p* adjust of <0.05 with expression values greater than 100 in either treatment. As a result, 320, 38, and 545 DEGs in E. coli BL21, S. cerevisiae AH109, and C. reinhardtii FACHB-479 were sorted out for further analyses. Significantly responsive genes were divided into several categories based on annotation results with online and local databases.

10.1128/mSystems.01189-21.2 FIG S2 Common enriched GOs in Escherichia coli BL21, Saccharomyces cerevisiae AH109, and Chlamydomonas reinhardtii FACHB-479. DEGs were considered the absolute value of log_2_ FC ≥ 1 and *p* adjust < 0.05. Download FIG S2, TIF file, 4.1 MB. Copyright © 2021 Chen et al. 2021 Chen et al. https://creativecommons.org/licenses/by/4.0/ This content is distributed under the terms of the Creative Commons Attribution 4.0 International license.

**FIG 2:**
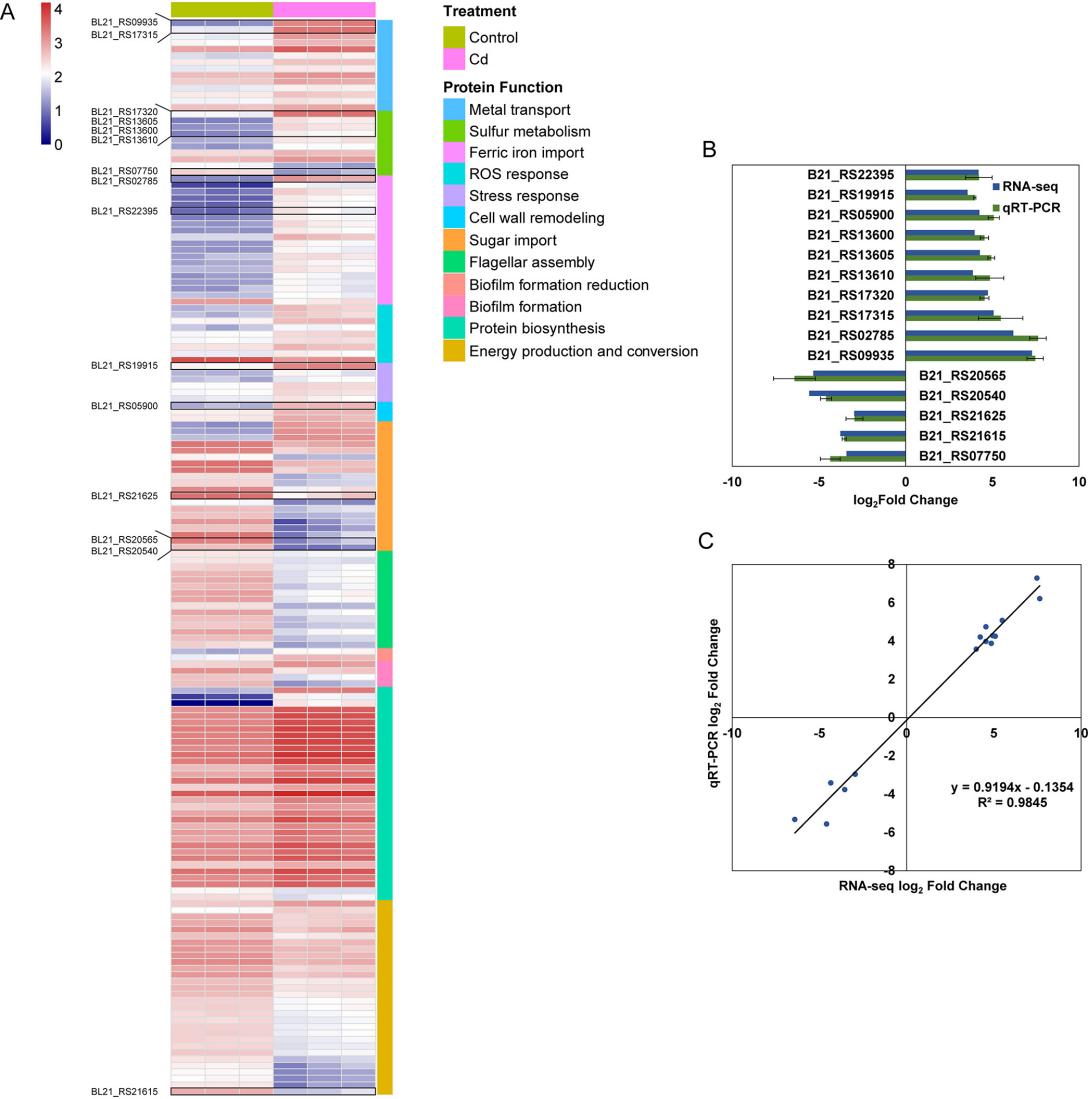
Typical differentially expressed genes (DEGs) and expression pattern in Escherichia coli BL21 under Cd stress. (A) A heatmap showing typical DEGs in E. coli BL21 under Cd stress. (B) qPCR verification of gene expression levels. qRT-PCR, quantitative reverse transcription-PCR. (C) Consistency analysis between RNA-seq and qPCR results. DEGs were selected according to the thresholds of the absolute value of log_2_ FC ≥ 1, *p* adjust < 0.05, and expression values greater than 100 in either Cd or control treatment.

In E. coli BL21, pathways of metal ion transport (35 genes, 10.9%; the ratio represents the number of the genes in this pathway to the total number of DEGs with expression values greater than 100), sulfur metabolism (10 genes, 3.1%), reactive oxygen species (ROS) response (9 genes, 2.8%), cell wall remodeling (3 genes, 0.9%), protein biosynthesis (33 genes, 10.3%), sugar transport (21 genes, 6.54%), flagellum assembly (15 genes, 4.7%), biofilm formation (6 genes, 1.9%), and energy metabolism (30 genes, 9.4%) were involved in the Cd response. Of the DEGs, almost all the genes involved in the first five pathways were upregulated, and the majority of the genes involved in the last four pathways were downregulated (Fig. 2A). Among the metal transporters, 4 genes were involved in Zn uptake (homologous gene of *zinT* and *znuABC*), and 21 genes were related to iron uptake. A Cu efflux system CusABCF (four genes), a cation efflux system AcrAB-TolC (three functional genes, one regulatory gene), and a ZntA homologous protein were involved in Cd detoxification. Under Cd stress, sulfur assimilation was mobilized: genes involved in sulfur import (two genes), sulfate and sulfite reduction (four genes), GSH biosynthesis (one gene), and cysteine/methionine (Cys/Met) dissociation (two genes) were upregulated (Fig. 2A).

**FIG 3:**
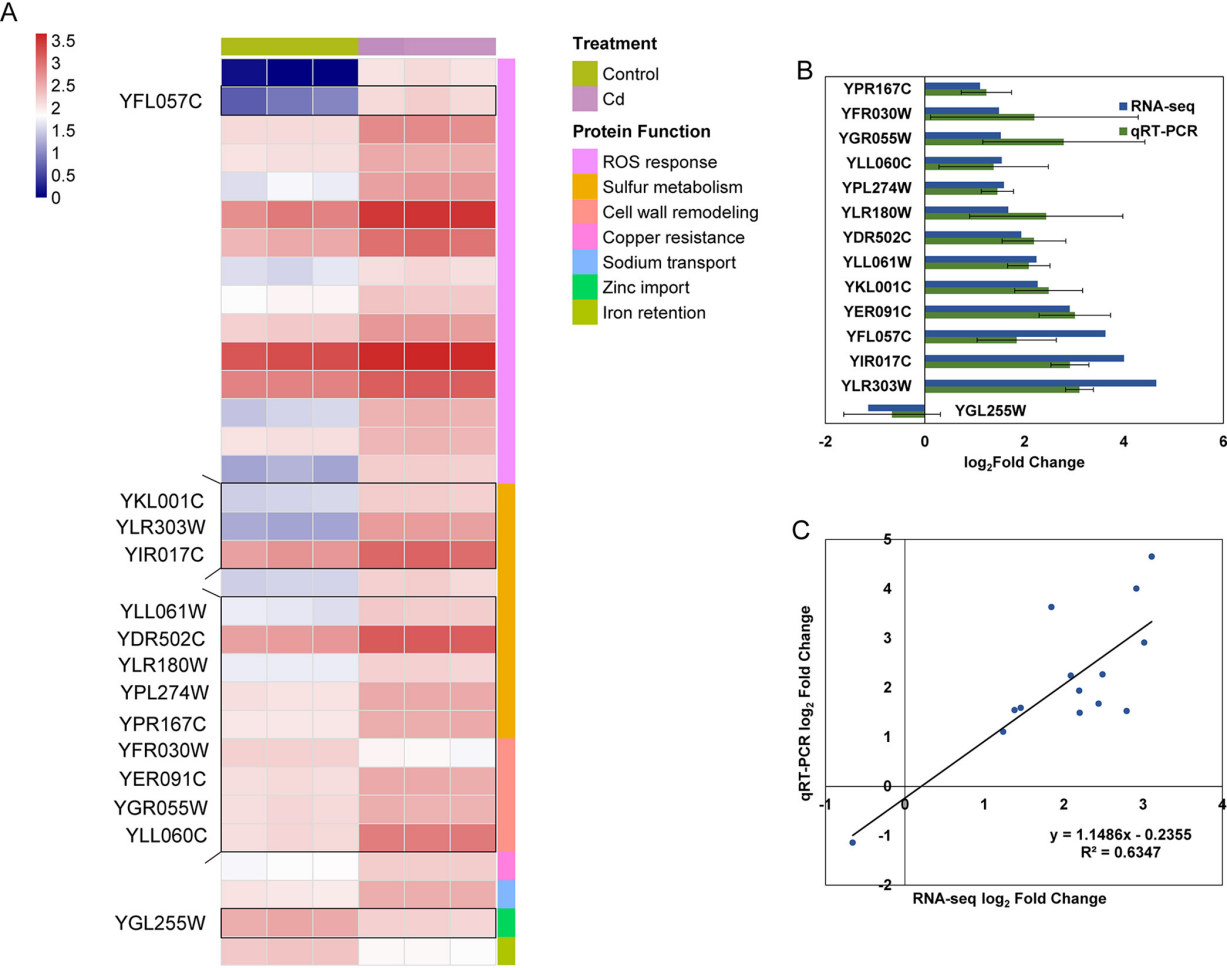
Typical differentially expressed genes (DEGs) and expression pattern in Saccharomyces cerevisiae AH109 under Cd stress. (A) A heatmap showing typical DEGs in S. cerevisiae AH109 under Cd stress. (B) qPCR verification of gene expression levels. (C) Consistency analysis between RNA-seq and qPCR results. DEGs are selected according to the thresholds of the absolute value of log_2_ FC ≥ 1, *p* adjust < 0.05, and expression values greater than 100 in either Cd or control treatment.

In S. cerevisiae AH109, sulfur metabolism (9 genes, 23.7%), ROS response (11 genes, 28.9%), cell wall remodeling (4 genes, 10.5%), metal ion transport (3 genes, 7.9%), and energy production and conversion (7 genes, 18.4%) were differentially expressed in response to Cd stress. Of these genes, genes involved in the first three pathways were upregulated due to Cd exposure, which was same as that in E. coli BL21, including several antioxidant enzymes such as thioredoxin (Trx), glutathione peroxidase (Gpx), peroxiredoxin (Prx), and antioxidant such as aldo-keto reductase and HSP. Genes involved in sulfur metabolism in S. cerevisiae AH109 mainly function in sulfate reduction (one gene), Cys/Met biosynthesis and transport (seven genes), and GSH biosynthesis (one gene). Genes encoding metal transporter and energy metabolism-related protein presented a different regulation pattern from E. coli BL21, for among three transporters participating in Zn (Zrt1), Fe (Fit1), and Na import, only the Na transport protein was activated. Several genes involved in energy production and conversion were upregulated in S. cerevisiae AH109 under Cd stress (Fig. 3A).

**FIG 4:**
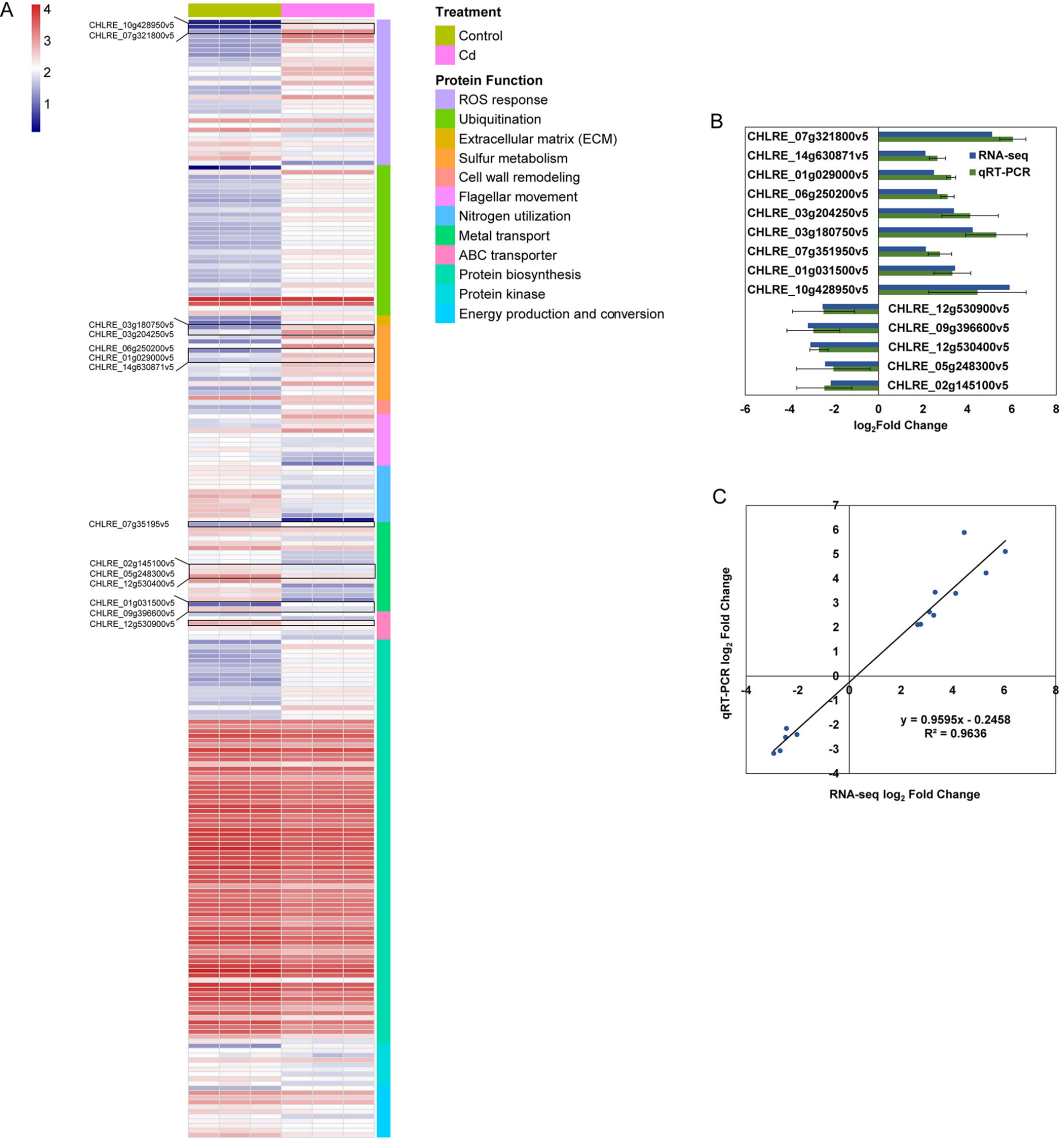
Typical differentially expressed genes (DEGs) and expression pattern in Chlamydomonas reinhardtii FACHB-479 under Cd stress. (A) A heatmap showing typical DEGs in C. reinhardtii FACHB-479 under Cd stress. (B) qPCR verification of gene expression levels. (C) Consistency analysis between RNA-seq and qPCR results. DEGs are selected according to the thresholds of the absolute value of log_2_ FC ≥ 1, *p* adjust < 0.05, and expression values greater than 100 in either Cd or control treatment.

Gene regulation under Cd stress was more complex in C. reinhardtii FACHB-479 than in E. coli BL21 and S. cerevisiae AH109 (Fig. 4A). Thirty-one genes encoding Trx (7 genes), superoxide dismutase (SOD, 2 genes), glutaredoxin (Grx, 2 genes), Prx (4 genes), HSP (10 genes), which are involved in ROS response, and about two-thirds of these genes were upregulated. Of these genes, all the HSP-encoding genes were upregulated, while the Prx-encoding genes were downregulated. Sixteen genes involved in intracellular sulfur metabolism were upregulated under Cd exposure, including genes encoding proteins participated in H_2_S (2 genes), Cys (2 genes), Met (10 genes), and GSH (3 genes) biosynthesis. Sixteen genes encoding metal transporters were downregulated, which were classified into the following: (i) Zn uptake transporter Zip (1 gene); (ii) Fe importers (1 gene) and the vacuolar sequestration transporters (2 genes), FTR1 and VIT1 (34); (iii) Ca importers (5 genes); and (iv) Cu efflux transporter (1 gene, *copA*). Two Cd transport proteins related to Cd (CHLRE_05g248300v5, homologous to *nmarp*) and GSH-Cd vacuolar sequestration (CHLRE_02g097800v5, homologous to *abcC1*) (35) were downregulated. Cell wall remodeling that was always found participated in Cd absorption, together with other related genes (three genes) were upregulated in C. reinhardtii FACHB-479, which was consistent with the other two organisms. Most of the flagellar assembly-related genes (7/11) were downregulated, which was consistent with E. coli BL21. Thirty-two DEGs involved in the ubiquitin-proteasome system, a main pathway for protein degradation, were found upregulated. In coping with Cd stress, C. reinhardtii FACHB-479 cells simultaneously reduced nitrogen utilization (12 genes) and its related protein biosynthesis (64 genes) (Fig. 4).

### Homolog protein families associated with Cd across the three organisms.

A cross-kingdom protein homolog comparison among all the DEGs was conducted. Twelve groups of homologous proteins were obtained, including the categories of metal ion transmembrane transporter, ABC transporter, sulfite reductase, phosphoglycerate dehydrogenase, aldo/keto reductase, HSP, metallopeptidase, iron-sulfur cluster assembly, fumarate reductase/succinate dehydrogenase, elongation factor, peptidyl-prolyl *cis*-*trans* isomerase, and ATP-dependent RNA helicase (Table 1).

**TABLE 1:** Twelve differentially expressed gene groups that were potentially homologous in E. coli BL21, S. cerevisiae AH109, and C. reinhardtii FACHB-479 under Cd stress

*E. coli*				*S. cerevisiae*				*C. reinhardtii*			
Gene_id	Log_2_ FC^*a*^	p adjust	Protein function	Gene_id	Log_2_FC	p adjust	Protein function	Gene_id	Log_2_ FC	p adjust	Protein function
B21_RS13195		<0.001	Glycine betaine/l-proline ABC transporter	YDR135C	0.81	<0.001	ABC type transporter YCF1	CHLRE_13g604150v5	0.60	<0.001	ABC transporter
B21_RS17315	5.07	<0.001	Zn/Cd/Hg/Pb-transporting ATPase	YDR039C	−0.57	<0.001	K/Na ion transmembrane transport	CHLRE_16g682369v5	−1.91	<0.001	Cu ion transmembrane transporter
B21_RS14345	0.28	0.03	d-3-Phosphoglycerate dehydrogenase	YIL074C	0.90	<0.001	Phosphoglycerate dehydrogenase SER33	CHLRE_16g689700v5	1.52	<0.001	Intracellular trafficking, secretion, and vesicular transport
B21_RS10695	−0.60	<0.001	Lactate dehydrogenase								
B21_RS13605	4.28	<0.001	Assimilatory sulfite reductase	YJR137C	1.02	<0.001	Sulfite reductase	CHLRE_16g693202v5	3.32	<0.001	Sulfite reductase
B21_RS15015	0.60	<0.001	2,5-Diketo-d-gluconic acid reductase	YDL243C	2.07	<0.001	Aryl-alcohol dehydrogenase	CHLRE_14g630400v5	0.81	<0.001	Aldo/keto reductase family
B21_RS01925	−0.87	<0.001	Aldo/keto reductase								
B21_RS15850	0.24	0.02	ATP-dependent zinc metalloprotease FtsH	YPR024W	0.51	<0.001	Metalloendopeptidase	CHLRE_17g716450v5	3.71	<0.001	Microtubule-severing ATPase
B21_RS12575	−0.27	0.02	Molecular chaperone HscA	YLL024C	0.51	<0.001	HSP70	CHLRE_14g613600v5	1.72	<0.001	DnaK
B21_RS03115	−0.45	0.02	Molecular chaperone HscC								
B21_RS00070	0.90	<0.001	Molecular chaperone DnaK								
B21_RS15770	1.29	<0.001	DEAD/DEAH box family ATP-dependent RNA helicase	YPL119C	0.67	<0.001	ATP-dependent RNA helicase	CHLRE_16g661900v5	0.56	<0.001	ATP-dependent RNA helicase
B21_RS21200	−0.61	<0.001	Fumarate reductase flavoprotein	YKL148C	1.06	<0.001	Succinate dehydrogenase flavoprotein subunit SDH1	CHLRE_17g696600v5	−0.55	<0.001	Fumarate reductase
B21_RS12840	−0.72	<0.001	l-Aspartate oxidase								
B21_RS17040	−0.32	0.003	Fe-S biogenesis protein NfuA	YKL040C	0.77	<0.001	Iron ion binding	CHLRE_18g748447v5	2.23	<0.001	Iron-sulfur cluster assembly
B21_RS16650	1.56	<0.001	Elongation factor	YJL102W	0.57	<0.001	Elongation factor	CHLRE_13g564950v5	1.17	<0.001	Elongation factor
B21_RS12810	−0.39	<0.001	Elongation factor								
B21_RS16775	0.76	<0.001	Peptidylprolyl isomerase	YDR155C	0.69	<0.001	Peptidyl-prolyl *cis*-*trans* isomerase	CHLRE_12g561000v5	−0.79	<0.001	Peptidyl-prolyl *cis*-*trans* isomerase
B21_RS02470	0.29	0.02	Peptidyl-prolyl *cis*-*trans* isomerase								

a FC, fold change.

### Validation of selected DEGs by qPCR.

The expression patterns for 43 (15 for E. coli BL21, 14 for S. cerevisiae AH109, and 14 for C. reinhardtii FACHB-479) DEGs identified by transcriptome sequencing (RNA-seq) were validated by quantitative PCR (qPCR) (Fig. 2B, 3B, and 4B). Although different algorithms were used in quantifying their expression levels, relatively comparable dynamics of gene expression patterns were observed by both approaches (Fig. 2B, 2C, 3B, 3C, 4B and 4C). Additionally, for each of the three organisms, RNA-seq data had a linear relationship with correlation coefficients of 0.985, 0.635, and 0.964 for E. coli BL21, S. cerevisiae AH109, and C. reinhardtii FACHB-479, respectively, indicating that the expression data obtained by RNA-seq were credible (Fig. 2C, 3C, and 4C). The qPCR-validated DEGs in E. coli BL21 were mainly involved in metal transport (B21_RS09935 and B21_RS17315), ferric iron import (B21_RS02785 and B21_RS22395), sulfur metabolism (B21_RS17320, B21_RS13610, B21_RS13605, B21_RS13600, and B21_RS07750), stress response (B21_RS19915), sugar import (B21_RS20540, B21_RS20565, and B21_RS21625), cell wall remodeling (B21_RS05900), and energy production and conversion (B21_RS21615) (Fig. 2A). In S. cerevisiae AH109, 11 selected DEGs (YLR303W, YIR017C, YKL001C, YLL061W, YDR502C, YLR180W, YPL274W, YLL060C, YGR055W, YFR030W, and YPR167C) encoded proteins for sulfur metabolism, one (YFL057C) encoded a protein for the ROS response, one (YGL255W) encoded a protein for zinc import, and one (YER091C) encoded a protein for methylation (Fig. 3A). The qPCR-validated DEGs in C. reinhardtii FACHB-479 were mainly involved in ROS response (CHLRE_10g428950v5 and CHLRE_07g321800v5), sulfur metabolism (CHLRE_03g180750v5, CHLRE_03g204250v5, CHLRE_06g250200v5, CHLRE_01g029000v5, CHLRE_14g630871v5, and CHLRE_12g525650v5), metal transport (CHLRE_07g351950v5, CHLRE_02g145100v5, and CHLRE_05g248300v5), copper oxidation (CHLRE_01g031500v5), septum formation (CHLRE_17g720350v5), and ABC transporter (CHLRE_12g530900v5) (Fig. 4A).

### An E. coli *ycf1* orthologous gene confers enhanced Cd tolerance in yeast.

**FIG 5:**
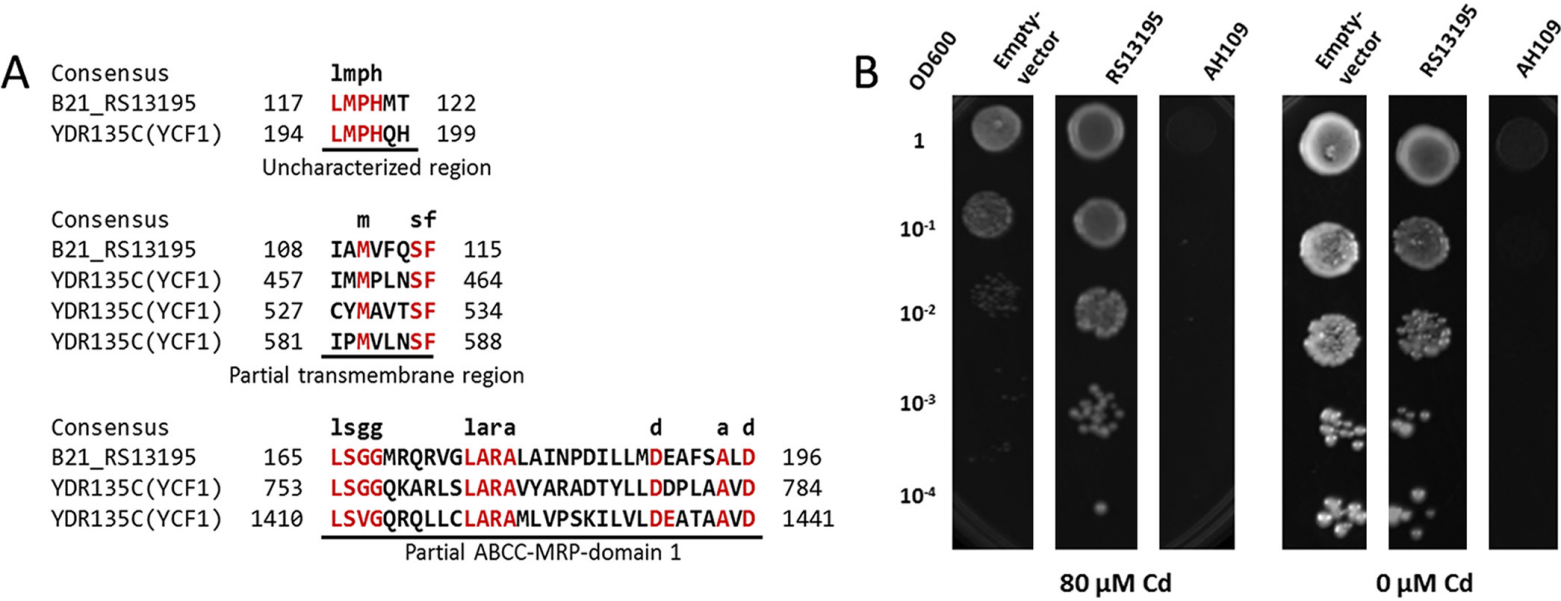
An E. coli *ycf1* orthologous gene conferred Cd tolerance in yeast. (A) Protein sequence alignment of homologous proteins classified as metal transporters in E. coli and S. cerevisiae. MRP, multidrug resistance-associated protein. (B) Drop assay for functional verification of Cd resistance in S. cerevisiae AH109 with B21_RS13195 overexpressed. Cells harboring empty PCEV vector and normal AH109 cells were used as negative controls in panel B.

To assess functional conservation of orthologous genes from different kingdoms associated with Cd stress, an *ycf1* homologous gene B21_RS13195 of E. coli BL21, which showed a similarity of 34.44% to the *ycf1* gene of yeast (Table 1 and Fig. 5), was selected and overexpressed in competent cells of S. cerevisiae AH109. Drop assay results showed that heterologous expression of the E. coli B21_RS13195 presented positive outcomes in Cd resistance (Fig. 5) with the transformant showing obviously better growth at 80 μM Cd than the control cells.

We also tried the overexpression of YDR135C (a YCF1 homologous gene in S. cerevisiae AH109) and CHLRE_13g604150v5 (a candidate ABC transporter in C. reinhardtii FACHB-479) in the E. coli BL21. Although the cDNAs of the two genes were successfully cloned, transformation of CHLRE_13g604150v5 in E. coli BL21 cells did not present obvious enhanced Cd tolerance (Fig. S3), neither did the construction of YDR135C transformant successfully.

10.1128/mSystems.01189-21.3 FIG S3 Functional verification of YDR135C and CHLRE_13g604150v5 in Escherichia coli BL21. (A) Full-length coding sequence amplification of YDR135C. (B) Full-length coding sequence amplification of CHLRE_13g604150v5. (C) Drop assay for functional verification of C. reinhardtii CHLRE_13g604150v5 under Cd stress in E. coli BL21. Download FIG S3, TIF file, 0.4 MB. Copyright © 2021 Chen et al. 2021 Chen et al. https://creativecommons.org/licenses/by/4.0/ This content is distributed under the terms of the Creative Commons Attribution 4.0 International license.

## DISCUSSION

A cross-kingdom comparative transcriptomic analysis was conducted for E. coli, S. cerevisiae. and C. reinhardtii in the present study to detect conserved pathways and genes for Cd stress response. Inhibition tests were conducted to determine the concentrations of Cd treatment for the three organisms which is critically important for a meaningful comparison. A total of 104.60 million paired-end reads were generated for the three organisms, resulting in overall 99%, 96%, and 95% total mapped reads after quality trimming. DEG analysis revealed that four comodulated pathways were associated with Cd stress, including ROS response, sulfur metabolism, cell wall remodeling, and metal ion transport among the tested species. *In vivo* expression patterns of 43 DEGs from the four pathways demonstrated a relatively comparable dynamic of gene expression patterns observed by both RNA sequencing and qPCR. Cross-kingdom protein sequence alignment led to the identification of 12 homologous protein clusters mainly functioning in ion transporter, ABC transporter, and sulfite reductase. In addition, an *ycf1*-like gene in E. coli was successfully expressed with enhanced Cd tolerance in yeast cells.

### Microbial Cd resistance is a global cellular response.

What makes a heavy metal-resistant microbe heavy metal resistant (36)? A conventional view of microbial metal resistance has long been established, which is mainly based on the exploration of dedicated metal resistance genes or gene clusters. Several Cd resistance determinants such as *cadA* in plasmid pI258 of Staphylococcus aureus (37), *czcA* in plasmid pMOL30 of Alcaligenes eutrophus (38), *nccA* carried by plasmid pTOM9 of Alcaligenes xylosoxidans (39), and *ycf1* in S. cerevisiae (40) have been found, and a series of homologous genes were also detected in other species (41–44). Characterization of these functional genes provided us a classical view of microbial Cd resistance, but it was restricted to looking only for specific genetic determinants (45, 46).

As a systems biology tool, transcriptomic analysis has greatly advanced our understanding on global response of gene regulation to environmental stress, including heavy metals (6, 47, 48). Through transcriptomic analysis, it was demonstrated that reactive oxygen generation, sulfur metabolites, DNA repair, and transport systems are involved in Cd stress response of Ganoderma lucidum (49). A Cu-resistant bacterium Cupriavidus gilardii CR3 also mobilized a spectrum of cellular activities, including sulfur metabolism, iron-sulfur cluster, and cell secretion systems for mediating Cu resistance (7). A transcriptomic analysis of tall fescue (Festuca arundinacea) showed that several metal transporters, GSH, and transcriptional factors were simultaneously induced by Cd (50). In our recent study, we also found that ROS response, cell wall remodeling, metal transport, and Cys synthesis were involved in the Cd stress response in P. ostreatus (5), and glycolysis/gluconeogenesis, pentose phosphate pathway and glycine metabolism were substantially induced by Cd treatment in Purpureocillium lilacinum strain YZ1 (51). With the information above, it is possible to establish an overview of global cellular responses to Cd stress systematically.

Here in this study, we further found that while classical metal transport genes were actively induced in the three tested species under Cd stress, a wider spectrum of cellular processes were actually involved. Top DEGs of all three species fell within the main categories of metal transport, sulfur metabolism, ROS response, and energy and protein synthesis. In fact, these cellular functions were reported in various organisms ranging from prokaryotes to plants to defend against Cd stress (see Table S2 in the supplemental material). Besides metal transporter genes, it seems that the top DEGs found in the current study respond universally against other stresses, such as heat, UV, and drought, from prokaryotic to eukaryotic organisms. A comparative transcriptomic analysis showed that heat and cold stress induced cell wall metabolism and HSP genes associated with the ROS response and that glutathione *S*-transferase-encoding genes are involved in the sulfur metabolism pathway of Isaria cateniannulata (52). ROS and redox changes may also be triggered as universal responses in *Cyanobacteria* toward heat, salt, hyperosmotic environment, and changes in light intensity (53). It is also well-known that energy metabolism, cell wall response, sulfate transport, and ROS response were commonly involved in salt, heat, and UV-B stresses of plants, such as Spinacia oleracea, Lycium ruthenicum, and Lotus japonicas (54–60). Taken together, it is now generally accepted that microbial Cd stress response includes not only dedicated/common metal transporters but also a wider spectrum of cellular processes that mostly are systems-level housekeeping.

10.1128/mSystems.01189-21.5 TABLE S2 Reported cell processes associated with the shared genetic modules of E. coli BL21, S. cerevisiae AH109, and C. reinhardtii FACHB-479 in response to Cd stress. Download Table S2, DOCX file, 0.03 MB. Copyright © 2021 Chen et al. 2021 Chen et al. https://creativecommons.org/licenses/by/4.0/ This content is distributed under the terms of the Creative Commons Attribution 4.0 International license.

### Isomorphy of microbial Cd stress response.

**FIG 6:**
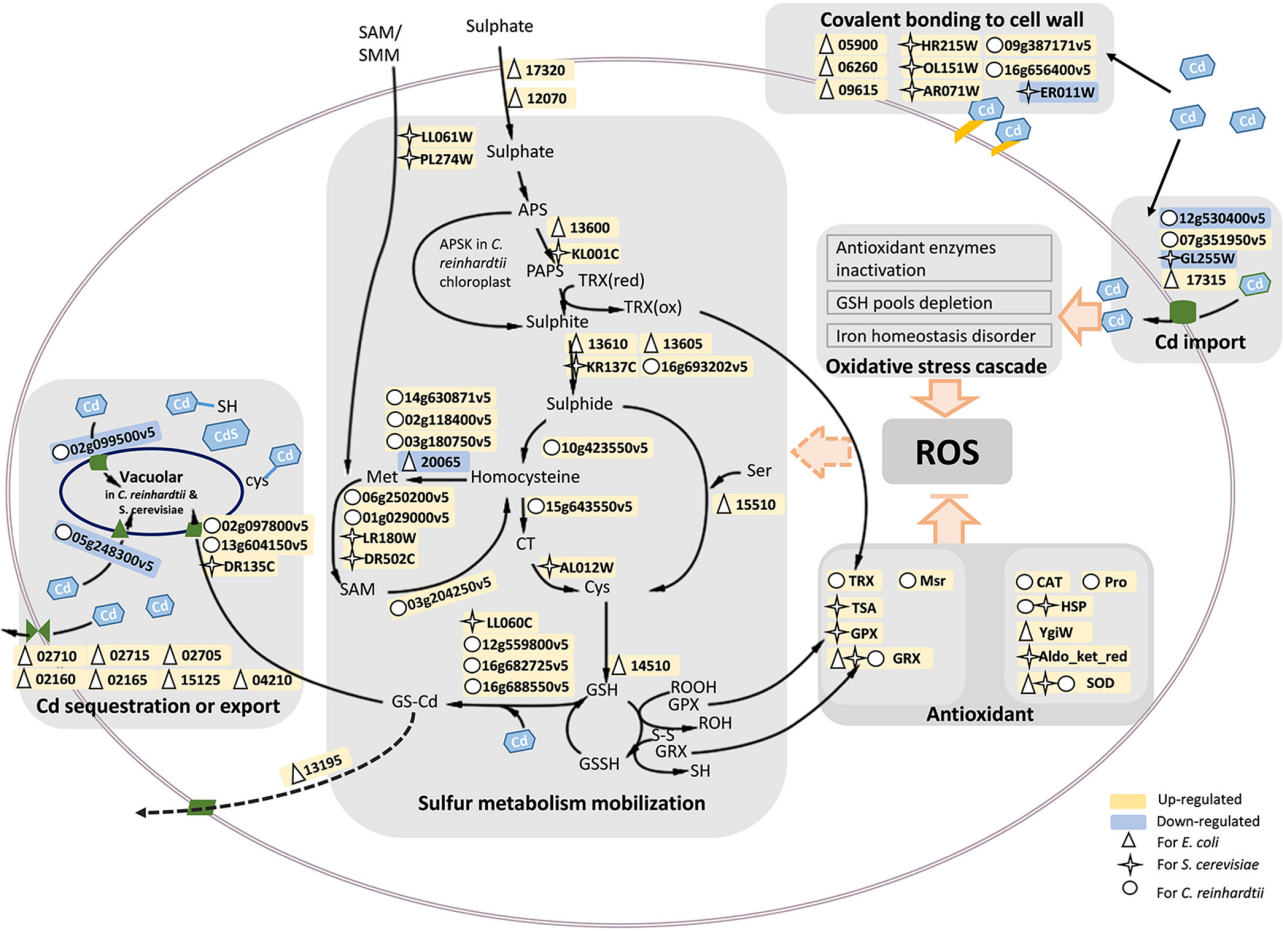
Schematic diagram summarizing the mechanisms involved in Cd stress response shared by the three organisms based on the comparative transcriptomic results. APS, adenosine 5′-phosphosulfate; APSK, ATP sulfurylase kinase; PAPSR, phosphoadenosine phosphosulfate reductase.

Comparative transcriptomics done in this study indicated an apparent isomorphy in microbial Cd stress responses. Forty-three selected DEGs that were verified through qPCR led to the formulation of a big picture of the shared pathways across the three species (Fig. 6). It is now well established that cellular processes are organized as a metabolic network at the systems level, which can be reflected by significant coexpression modules (61, 62). From our results, four significant coexpression modules were detected across the three microbial species, including cell wall remodeling, ROS response, sulfur metabolism, and metal transport (Fig. 6).

DEGs associated with cell wall remodeling may play a role in covalent Cd binding as found in this study and elsewhere (5, 58, 63, 64), probably through the modification of cell wall chemistry. Besides cell wall protection, transmembrane Cd importers were found actively involved in Cd stress response. Interestingly, Zn importer genes were universally induced by Cd stress in the three species (Table 1), including *zinT* and *znuABC* in E. coli (65), *zrt1* in yeast (66), and *zip* genes in C. reinhardtii (67). Considering that Cd is chemically similar to Zn, and both of them belong to the IIB transition elements, it is probably common in microbial species that Cd is imported via the Zn channels. It was reported that with ZnuABC as companions, ZinT plays an apparent role in Cd import (26), and ZRT1 participates in Cd uptake in S. cerevisiae (68). Deletion or inactivation of *zrt1* in wild-type yeast led to a substantial decrease in Cd uptake (69, 70). Though evidence for C. reinhardtii is still not available, the role of *zip* in Cd uptake in other plants has been well characterized (71, 72).

Intracellular Cd can induce an oxidative stress cascade, for all known tested species, including the ones used in this study (73, 74). Due to the high affinity between Cd and sulfur (24), intracellular Cd can easily bind to thiol-rich GSH (75, 76), resulting in GSH depletion (30). Additionally, Cd competition for S^2−^ in Fe-S cluster accelerates the Fenton reaction (77). In the current study, Cd exposure results in the induction of antioxidation reaction, as it was demonstrated by the significant upregulation of GSH synthesis pathway in all three organisms (Fig. 2 to 4). At the same time, we demonstrated upregulation of some thiol enzymes (Trx, Grx, Gpx, TsA, and Msr), non-thiol-enzymes (SOD and catalase) and antioxidants (HSP, proline, and aldo/keto reductase) in all three species that can scavenge ROS (Fig. 6) (47, 78–80).

Under Cd stress, some genes related to sulfate and *S*-adenosylmethionine/*S*-methylmethionine (SAM/SMM) uptake, sulfur assimilation, and sulfur-containing amino acid synthesis were upregulated in all three species (Fig. 6). The production of H_2_S, Cys, and GSH may effectively chelate Cd and were involved in reducing Cd toxicity (81–84). Exactly, in our study, the genes encoding glutathione transferase (GST) that were responsible for conjugation of Cd with GSH (85) were significantly enriched in S. cerevisiae (YLL060C) and C. reinhardtii (CHLRE_16g682725v5, CHLRE_12g559800v5, and CHLRE_16g688550v5) (Fig. 6). Additionally, genes involved in metal efflux and sequestration such as *ycf1*, *nramp* (86), *vit* (34), *cusABCF* and *acrABC* (87, 88) homologs were enriched in our study (Fig. 6), which may function in Cd detoxification.

### Important Cd-responding genes across the three species.

Twelve groups of common response orthology proteins in the three species were obtained by comparative transcriptomic analysis (Table 1). It was found that besides metal transporters, more proteins associated with basic metabolism of substance synthesis/energy were involved. Of these proteins, hydroxypyruvate reductase, phosphoglycerate dehydrogenase, elongation factor, and RNA helices peptidyl-prolyl *cis-trans* isomerase are well-known proteins for amino acid (Cys) and protein synthesis (89–91). Fumarate reductase, succinate dehydrogenase flavoprotein, and iron-sulfur cluster are the basic components of the respiratory chain for energy metabolism (92). Actually, substance synthesis and energy metabolism have been found to participate in Cd response in a number of other organisms (5, 93–95). Generally, it can now be concluded that microbial Cd response involves global systems activities, which include not only dedicated functional modules particularly metal transport but also many other basic metabolic pathways.

Experimental validation of orthology of one of the above-mentioned proteins was performed tentatively by expressing a novel *ycf1*-like gene of E. coli BL21 in yeast. Yeast Ycf1 is a well-characterized Cd-GSH-importing transporter on vacuole membrane (23), while the function of E. coli *ycf1* homolog encoded by B21_RS13195 remains unknown. Gene B21_RS13195 was substantially induced by Cd stress in the comparative transcriptomic study, and sequence analysis indicated that it might be a transporter gene and shared a high amino acid sequence similarity to yeast Ycf1. Together with the experimental results of its overexpression in yeast (Fig. 5), E. coli B21_RS13195 seems to be a novel Cd resistance transporter gene that homologous to yeast *ycf1*. Previous studies showed that Ycf1 could sequester GSH-Cd composition into vacuoles and improved Cd resistance of S. cerevisiae (23). As discussed, modern omics tools possess great potential in discovering novel genes, which could also contribute to knowledge on prokaryotic Cd resistance (96–98). In a recent study, comparative genomics of Cd-resistant strains through evolution in the laboratory also led to the discovery of two novel Cd resistance genes in E. coli, *htpX* encoding an integral membrane heat shock protein and *gor* encoding glutathione reductase (99). Either the B21_RS13195 gene or the *htpX/gor* gene is not closely related to known genetic determinants for Cd resistance like *cad* or *czc*, which necessitates the use of systems biology tools. We also tried to express *ycf1* and CHLRE_13g604150v5 (a candidate ABC transporter in C. reinhardtii) in the E. coli strain, yet it was not successful. It may mainly be due to the simplicity of prokaryotic cells that cannot support the complex regulation processes of eukaryotic genes (see Fig. S3 in the supplemental material). Phylogenetic analysis of eukaryotic metal transporters showed that higher organisms tend to develop a more complex system for metal homeostasis (8). Yeast Ycf1 is a typical Cd efflux ATPase, whose function may rely on the presence of other proteins, such as copper chaperone for copper zinc superoxide dismutase 1 (CCS1) that may be absent in E. coli (100). In addition, successful expression of eukaryotic genes in prokaryotic cells is still challenging and may require different recognition sites for RNA polymerase and a wide spectrum of posttranslational machineries (101, 102), though prokaryotic gene resources have been commonly used in genetic engineering of eukaryotic organisms (4, 103, 104). Taken together, the results indicate that comparative transcriptomics possess a great potential in the discovery of novel genes, resulting in a novel *ycf1*-like gene discovered in E. coli and exhibiting a high level of Cd resistance in the yeast host cells. Cross-expression of the detected orthologous genes deserves a further study, which is necessary for understanding the evolutionary relationship of the shared pathways/functional modules for microbial Cd stress response.

## MATERIALS AND METHODS

### Strains and inhibition test.

Escherichia coli strain BL21 (Invitrogen, USA), Saccharomyces cerevisiae strain AH109 (Clontech, USA) and Chlamydomonas reinhardtii strain FACHB-479 (GDMCC, China) were used in this study. Cultures of the three strains were started by activating frozen stocks and streaking onto an agar plate under required conditions. For strain activation, a single colony was chosen, streaked, and cultured at the optimum condition. The procedure was repeated until the strain recovered normal growth. Then, a single colony of E. coli BL21 from a Luria-Bertani (LB) agar plate was inoculated into 5 ml liquid LB medium and incubated with shaking overnight at a speed of 180 rpm at 37°C in dark. The cell suspension was diluted for initiating the growth curve test. The density of the suspension was measured every 30 min. S. cerevisiae AH109 was inoculated into liquid yeast extract-peptone-dextrose (YPD) medium at 28°C and shaked at 180 rpm in dark. C. reinhardtii FACHB-479 was inoculated in liquid Bristol’s medium with a 16-h/8-h day/night cycle at 25°C. The density (optical density at 600 nm [OD_600_]) of the algae suspension was measured once a day.

A Cd treatment and a no-Cd exposure control were used in the current study for each species. The concentrations used in the Cd treatment were determined by inoculating the isolates in liquid medium with various concentrations of Cd. The concentration of Cd that resulted in a 30% to 40% reduction of the logarithmic growth rate compared to that of the control was chosen as the Cd treatment concentration for subsequent experiments, since at this level of inhibition we assumed that cellular Cd stress would be induced. E. coli BL21 was treated with Cd concentrations of 100, 200, 400, 600, or 800 μM. S. cerevisiae AH109 was treated with Cd concentrations of 25, 50, 100, 200, or 400 μM. C. reinhardtii FACHB-479 was treated with 4, 8, 12, 16, or 20 μM Cd treatment concentration.

### Transcriptomic sequencing.

Cells of C. reinhardtii FACHB-479, S. cerevisiae AH109, and E. coli BL21 were collected by centrifugation after incubation, with three replicates for each treatment. Total RNA was extracted from samples using TRIzol reagent (Invitrogen, Carlsbad, CA, USA) following the manufacturer’s instructions. RNA samples were immediately purified using RNeasy MinElute Cleanup kit (Qiagen, MD, USA). The quantity of the isolated RNA was examined using a ND 1000 nanodrop spectrophotometer (Thermo Scientific, Waltham, MA, USA) and verified through agarose gel electrophoresis. The integrity of total RNA was assessed using Agilent 2100 (Agilent Technologies, Santa Clara, CA, USA). RNA integrity numbers (RINs) from 1 to 10 were assigned to each sample to indicate its integrity or quality. In total, 2-μg RNA samples with a concentration greater than 100 ng/μl and RINs ranging from 7 to 10 were used for RNA library preparation, with the TruSeq Stranded mRNA Library Prep kit (Illumina, San Diego, CA). Results showed that all sample RINs were above 8.5, which qualified for cDNA library preparation. The cDNA library was constructed using the extracted mRNA with the Truseq RNA sample prep kit (Illumina, San Diego, CA, USA). Sequencing was conducted using the Illumina HiSeq 4000 platform (Illumina, San Diego, CA, USA) provided by Majorbio Bio-Pharm Technology Co., Ltd., Shanghai, China.

### Reference mapping and RNA-seq analysis.

Eighteen sequence assemblages containing 403.76, 363.09, and 104.60 million raw reads for E. coli BL21, S. cerevisiae AH109, and C. reinhardtii FACHB-479, respectively, were generated by Illumina sequencing. Adapters were trimmed off using SeqPrep (omicX, Le-Petit-Quevilly, France), and raw data were subsequently passed through quality trimming by using Sickle (omicX, Le-Petit-Quevilly, France). Reads with incorrectly called bases toward the 3′ end and 5′ end were trimmed off. Phred score (Q30), GC content, and sequence duplication level of the clean data were calculated. The high-quality clean data obtained were used in reference mapping.

The reference genomes for C. reinhardtii FACHB-479 (105), S. cerevisiae AH109, and E. coli BL21 were downloaded from National Center for Biotechnology Information (NCBI) with the identity (ID) of 147, 15, and 167, respectively. To ensure the accuracy of our analysis, no more than five mismatches were allowed in the alignment. The alignment data were utilized to calculate the distribution of reads on the reference genes and to perform the coverage analysis. Outputs of the sequence alignment containing the aligned reads and mapping information were used for the downstream analyses. Data analysis was performed on the online platform of Majorbio I-Sanger Cloud Platform (Majorbio, Shanghai, China).

### Differential gene expression analysis.

Transcripts per million (TPM) were used to describe the expression level of genes obtained from genome mapping. Differentially expressed genes (DEGs) were obtained by comparing the control and Cd treatment groups. Genes were described as up- or downregulated in Cd treatment groups. The absolute value of log_2_ fold change (FC) ≥1 and *p* adjust < 0.05 were selected as stringent thresholds to choose prominent differential gene activity.

### Functional annotation.

The identified DEGs were annotated to Gene Ontology (GO) and Kyoto Encyclopedia of Genes and Genomes (KEGG) pathways and then subjected to enrichment analysis at the threshold of corrected *P* < 0.05. The protein functional category for DEGs was assigned by blasting against UniprotKB/Swiss-Prot (swissprot) database. Selective DEGs with expression value greater than 100 were subsequently subjected to heatmap creation, qPCR validation, and common response gene/pathway analysis.

The protein sequences of annotated genes from the three species were merged as a local sequence. Cross-kingdom comparison of homologous protein was conducted using blastp against the local database (106). Only the matches having an expectation value (E value) of ≤0.001 and identity of ≥30% were considered homologous proteins (29, 107).

### Validation of selected DEGs by qPCR.

Forty-three genes (15 for E. coli BL21, 14 for S. cerevisiae AH109, and 14 for C. reinhardtii FACHB-479) derived from significantly differentially expressed genes (log_2_ fold change [FC] ≥1 and *p* adjust < 0.05) were associated with ion transport and sulfur metabolism pathways were selected for quantitative determination of their *in vivo* expression under Cd stress. Forward and reverse primers for amplifying the genes selected were designed using the web-based primer design tool Primer-BLAST on NCBI. Where possible, primers were designed to possess an optimum annealing temperature of 60°C, a GC content of 40% to 60%, an amplicon length of 150 to 200 bp, and a primer length of 20 to 24 bp. Candidate primers were compared against the reference genomes of the three organisms predicted coding DNA sequences (CDS) to select the gene-specific primers (see Table S3 in the supplemental material).

10.1128/mSystems.01189-21.6 TABLE S3 Primers for qPCR analysis. Download Table S3, DOCX file, 0.02 MB. Copyright © 2021 Chen et al. 2021 Chen et al. https://creativecommons.org/licenses/by/4.0/ This content is distributed under the terms of the Creative Commons Attribution 4.0 International license.

DNase-treated RNA (2 μg) was used to synthesize first-strand cDNA using PrimeScript reverse transcription (RT) reagent kit with gDNA Eraser (TaKaRa Biomedical Technology Co., Ltd., Beijing, China). The cDNA was diluted 20 times, and a 2.4-μl aliquot was used in a 15-μl quantitative PCR (qPCR) reaction mix: 7.5 μl of SYBR green qPCR Master Mix (Vazyme Biotech Co., Ltd., Nanjing, China), 0.6 μl of 10 μM forward/reverse primer (Thermo Fisher Scientific Inc., MA, USA), and 3.9 μl of nanopure water. Real-time qPCR amplification and detection were conducted using CFX Connect real-time PCR system (Bio-Rad Laboratories, Inc, CA, USA) and the following protocol: a single cycle of 10 min at 95°C, followed by 40 cycles, with 1 cycle consisting of 15 s at 95°C and 30 s at 60 or 61°C. Relative gene expression was measured using the control group as the calibrator. No reverse transcriptase and no-template negative controls were included in every PCR amplification. Each sample was represented by two independent total RNA isolations converted into two separate cDNAs. Each cDNA sample was included using three technical replicates for PCRs. The target gene expression was normalized to that of the internal reference genes of each of the three organisms using the 2^−ΔΔCT^ method (the comparative threshold cycle [*C_T_*] method) (108).

### Overexpression of an E. coli Cd transporter gene in S. cerevisiae.

Through homologous protein screening among the three organisms, a group of potential Cd transporter genes were found possessing conservative sequence. We tested the overexpression and Cd resistance function of the E. coli BL21 copy in the yeast S. cerevisiae AH109, as well as S. cerevisiae AH109 and C. reinhardtii FACHB-479 copy in E. coli BL21 using a drop assay. Primers for full-length CDS amplification were designed (Table S4). The cloned genes were subsequently ligated to expression vector after adding restriction sites (Table S4). For yeast cell transformation, combined plasmids were introduced to competent cells of S. cerevisiae AH109 using a lithium acetate-based method (109), and a drop assay was conducted to examine the Cd sensitivity of the transformants. Vector pCEV-G1-Km under the PGK1 promoter (110) was used for S. cerevisiae AH109, and positive transformants were selected on solid YPD medium supplied with 300 μg/ml Geneticin (G418). The selected positive clones were transferred to liquid YPD medium with 300 μg/ml G418. Cells in suspension were subsequently collected by centrifugation and serially diluted (OD_600_ of 1.0, 10^−1^, 10^−2^, 10^−3^, and 10^−4^) with sterilized water. Five microliters of each dilution was spread onto the YPD plates containing 300 μg/ml G418 and 80 μM CdCl_2_ at 28°C for 5 days. Cells harboring empty pCEV-G1-Km and inoculated on YPD plates without Cd incubation were used as the negative control. Vector PET-28a (Novagen, Germany) was used for E. coli transformation, and positive clones were selected on solid LB plates with 50 μg/ml kanamycin. Drop assay with a serially diluted positive cell (OD_600_ of 1.0, 10^−1^, 10^−2^, 10^−3^, and 10^−4^) was performed on LB plates with 50 μg/ml kanamycin, 600 μM Cd, and 200 μM isopropyl-β-d-thiogalactopyranoside (IPTG). The colonies harboring empty pET-28a were used as a negative control. Cadmium concentrations used for drop assay for yeast and E. coli were screened and selected when the growth of transformants and control were clearly distinguished. Results of at least three biological replicates were averaged for all experiments.

10.1128/mSystems.01189-21.7 TABLE S4 Primers for yeast expression vector construction. Download Table S4, DOCX file, 0.01 MB. Copyright © 2021 Chen et al. 2021 Chen et al. https://creativecommons.org/licenses/by/4.0/ This content is distributed under the terms of the Creative Commons Attribution 4.0 International license.

### Statistical analysis.

All comparisons were subjected to one-way analysis of variance (ANOVA) using SPSS 16.0 software (version 16.0, IBM, New York, NY, USA). Means separation was conducted by using Duncon’s multiple range test, with *P* ≤ 0.05 considered significant. Figures were generated by R ver. 3. 6.1.

### Data availability.

RNA-Seq raw data were deposited in SRA (Sequence Read Archive) with BioProject accession numbers PRJNA692506, PRJNA692576, and PRJNA693088.
