# Supplementary figures and images for: Cross-Kingdom Comparative Transcriptomics Reveals Conserved Genetic Modules in Response to Cadmium Stress
Source: mSystems. 2021 Dec 7;6(6):e01189-21. doi: 10.1128/mSystems.01189-21 (PMC8651089; doi:10.1128/mSystems.01189-21)

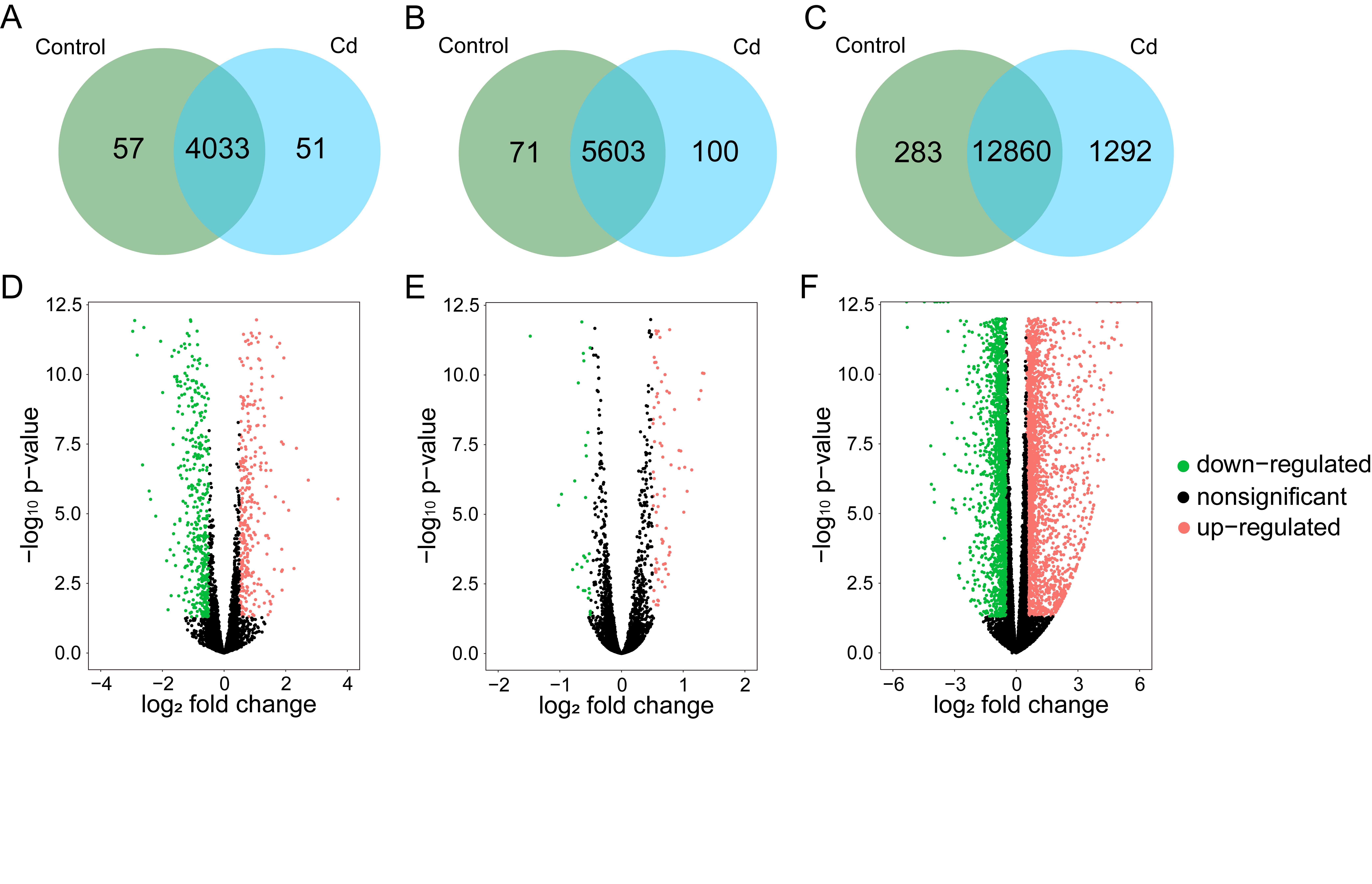

Supplement: FIG S1 [file msystems.01189-21-sf001.tif]

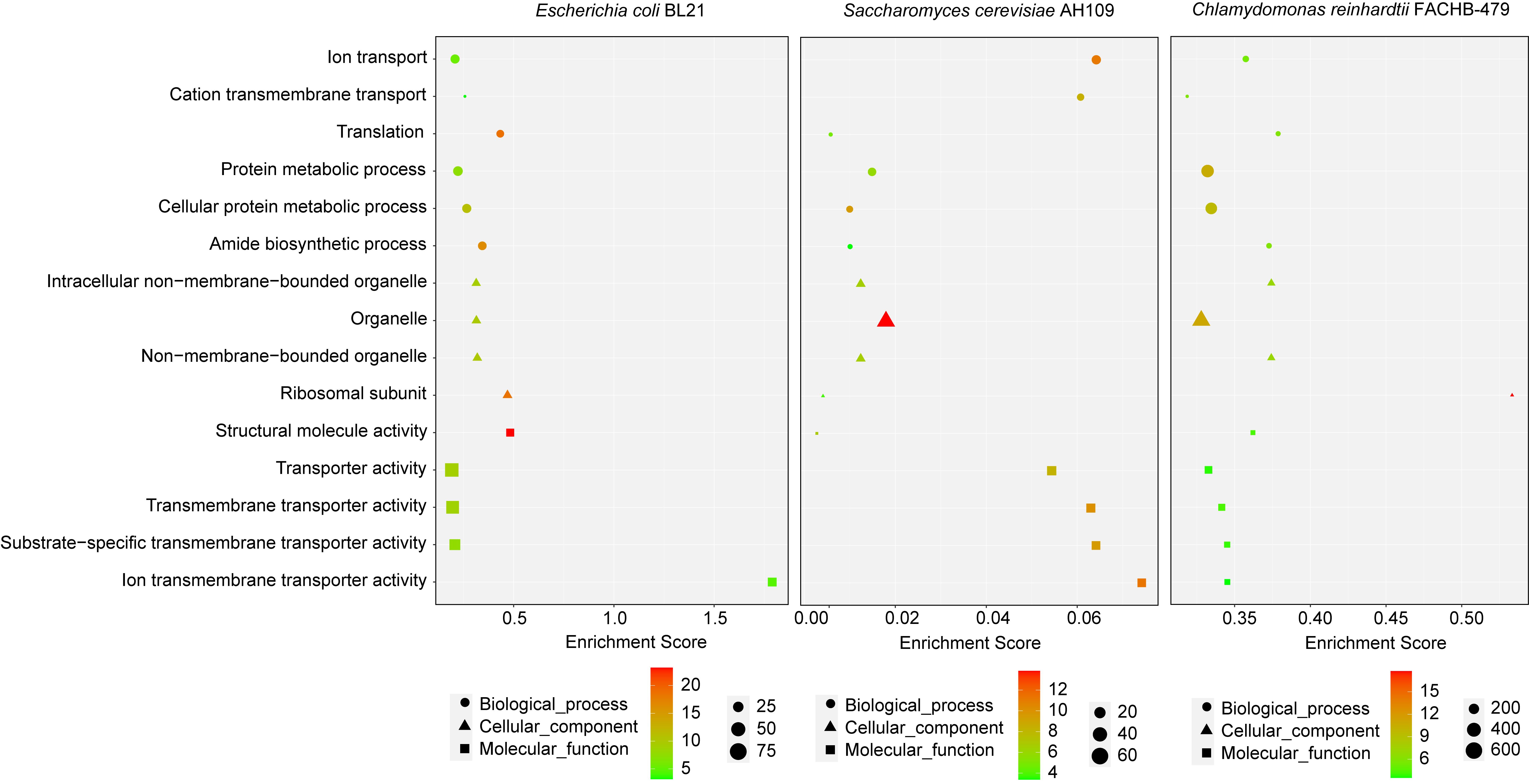

Supplement: FIG S2 [file msystems.01189-21-sf002.tif]

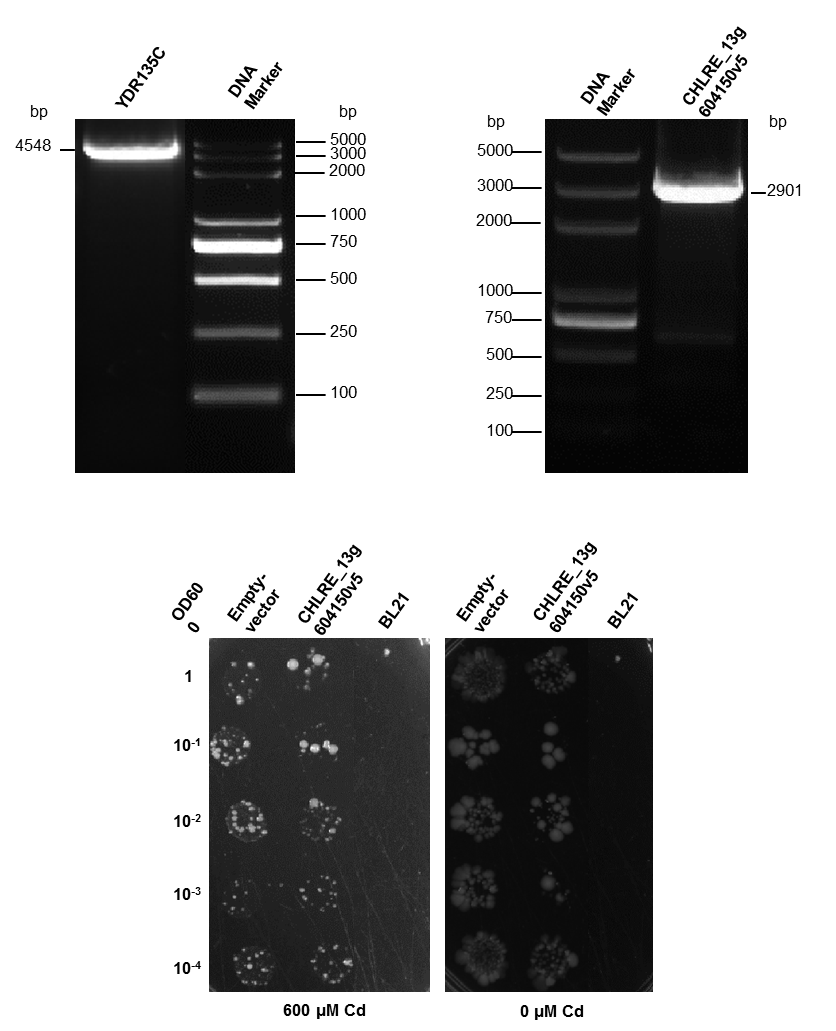

Supplement: FIG S3 [file msystems.01189-21-sf003.tif]
