# Supplementary material for: Cross-Kingdom Comparative Transcriptomics Reveals Conserved Genetic Modules in Response to Cadmium Stress
Source: mSystems. 2021 Dec 7;6(6):e01189-21. doi: 10.1128/mSystems.01189-21 (PMC8651089; doi:10.1128/mSystems.01189-21)
Supplement: TABLE S1 [file msystems.01189-21-st001.docx]

|  |  |  | ***Escherichia coli***  **BL21** | ***Saccharomyces cerevisiae*** **AH109** | ***Chlamydomonas reinhardtii* FACHB-479** |
| --- | --- | --- | --- | --- | --- |
| **G+C (%)** |  |  | 51.35 | 42.83 | 63.86 |
| **Q30 (%)** |  |  | 95.06 | 96.38 | 94.46 |
| **Mapped reads** |  |  | 16,971,332 | 57,484,390 | 63,176,350 |
|  | Uniquely mapped reads |  | 16,699,701 | 52,623,688.33 | 60,262,107.67 |
|  |  | CDS (%) | 93.50 | 98.98 | 78.80 |
|  |  | 3'UTR (%) | N/A | 0.08 | 15.98 |
|  |  | 5'UTR (%) | N/A | 0.00 | 4.54 |
|  |  | Intro (%) | N/A | 1.07 | 0.53 |
|  |  | Intergenic (%) | N/A | 0.01 | 0.17 |
|  | Multiple mapped reads |  | 271,631 | 4,860,701.67 | 2,914,242.33 |
| **Total reads** |  |  | 17,131,120.67 | 59,669,842.00 | 66,501,326.33 |

^z^ The references genomes for *C. reinhardtii* FACHB-479 (Witman et al., 2007), *S. cerevisiae* AH109, and *E. coli* BL21 were downloaded from National Center for Biotechnology Information (NCBI) with the ID of 147, 15, and 167, respectively
