# Supplementary material for: Cross-Kingdom Comparative Transcriptomics Reveals Conserved Genetic Modules in Response to Cadmium Stress
Source: mSystems. 2021 Dec 7;6(6):e01189-21. doi: 10.1128/mSystems.01189-21 (PMC8651089; doi:10.1128/mSystems.01189-21)
Supplement: TABLE S2 [file msystems.01189-21-st002.docx]

| **Species** | **ROS response** | **Sulfur metabolism** | **Cell wall response** | **Transporter** | **Reference** |
| --- | --- | --- | --- | --- | --- |
| *Bacillus cereus* | SOD increased | GSH elevated | Bound Cd by carboxyl | ABC transporters | (1-3) |
| *B. subtilis* |  | Bacillithiol increased | Bound Cd by carboxyl | Mn^2+^ transporter, CzcD, YvgW, CadA | (4-10) |
| *E. coli* | CAT increased | GSH, Cys and FeS increased | Adsorb one third of Cd | ZntA, CzcCBA, CzcD, ZinA | (11-18) |
| *Pseudomonas sp.* | Induced AOEs | Induce various of MT | Mainly located on cell wall | CzrCBA, CadA, CadR | (19-23) |
| *A. ferrooxidans* | GSH increased | Enhance sulfur uptake and sulfur assimilation |  | RND transporters and cation diffusion facilitators | (24-26) |
| *N. crassa* | Peroxidase increased | Cds accumulation | 36% Cd bound to cell wall | Tzn2 | (27-29) |
| [*S. cerevisiae*](http://med.wanfangdata.com.cn/Paper/Detail/PeriodicalPaper_PM11078740) | Induce Ahp, Trx, Tsa, Ccp1p, CAT, SOD, Hsp | Cysteine, GS and sulfate assilimated related enzymes increased | Activate cell wall construction and morphogenesis | Ycf1, BPT1, Smf1, Cot1, Zrc1, ZRT1, CadA, Alrp, Opt/Hgt, PCA1p | (30-43) |
| *Trichoderma harzianum* | Upregulated Aoes | Induce GSH and its precursor’s metabolism | Downregulated CAZymes | ABC transporters | (44) |
| *Aspergillus* sp. | SOD, CAT, and GR increased | Thiol contents increased | Cd biosorption mainly by chitosan | NmeA | (45-48) |
| *P. ostreatus* | Enhance activities of SOD, POD, and CAT | Cys-rich proteins upregulated | Significantly induced cell wall remolding | MFS, CAX, MATE | (49, 50) |
| *G. lucidum* | Strongly induced dyp-type Prx | Enhance sulfur metabolites | Cell wall biosynthesis | K transporter and sec1-like transporter | (51) |
| *C. reinhard*tii | GPX5 unregulated | GSH metabolism related genes unregulated | Less Cd accumulated in cell wall-less strain | Ca-transporting ATPase, CrCds | (52-54) |
| *C. vulgaris* | Proline accumulated  Induce SOD, CAT, PRX | GSH accumulated | Bound 50% of the cellular Cd^2+^ | Slow uptake Cd by member transporter | (54-57) |
| *A. thaliana* | Enhance activity of SOD and ASP | H_2_S, Cys, GSH, GS, MT accumulated, sulfate transport increased | Hemicellulose 1 fix Cd | AtHMA4, AtHMA3, AtCAX, AtNRAMP3, AtMRP3, AtIRT1, AtOPT6, VIT1 | (39, 58-70) |
| *T. caerulescens* | GR, SOD increased | GSH and sulphate metabolism increased | Hold most of Cd in cell wall | ZNT1, IRT1, HMA4, CAX3, CAX7, TgMTP1, TcOPT3 | (71-77) |
| *Oryza sativa* | SOD, MGO and MDA increased | Endogenous H_2_S accumulated | Alterations of pectin and hemicellulose contents | OsHMA2, OsNRAMP5 | (78, 79) |
| *Hordeum vulgare* | Enhance activity of CAT2 and SOD | Enhance activity of GST | Cell wall remodeling | Enhance cation ion transport | (80) |

Notes: Ahp: alkyl hydroperoxidase, Tsa: thioperoxidase, Ccp1p: cytochrome peroxidase, CAT: Catalase, GR: glutathione reductase, ASP: ascorbate peroxidase, MDA: malondialdehyde, MGO: methylglyoxal, GST: glutathione S-transferases, MATE: multidrug and toxic compound extrusion, AOEs: antioxidant enzym

**References**

1. Behera M, Dandapat J, Rath CC. 2014. Effect of heavy metals on growth response and antioxidant defense protection in *Bacillus cereus*. J BASIC MICROB 54:1201-1209.

2. Wang C, Liu Z, Huang Y, Zhang Y, Wang X, Hu Z. 2019. Cadmium-resistant rhizobacterium *Bacillus cereus* M4 promotes the growth and reduces cadmium accumulation in rice (*Oryza sativa* L.). Environ Toxicol Phar 72:103265.

3. Huang F, Guo CL, Lu GN, Yi XY, Zhu LD, Dang Z. 2014. Bioaccumulation characterization of cadmium by growing *Bacillus cereus* RC-1 and its mechanism. Chemosphere 109:134-142.

4. Burke BE, Pfister RM. 1986. Cadmium transport by a Cd^2+^-sensitive and a Cd^2+^-resistant strain of *Bacillus subtilis*. Can J Microbiol 32:539-542.

5. Laddaga RA, Bessen R, Silver S. 1985. Cadmium-resistant mutant of *Bacillus subtilis* 168 with reduced cadmium transport. J Bacteriol 162:1106-1110.

6. Guffanti AA, Wei Y. 2002. An antiport mechanism for a member of the cation diffusion facilitator family: divalent cations efflux in exchange for K^+^ and H^+^. Mol Microbiol 45:145-153.

7. Solovieva IM, Entian K-D. 2002. Investigation of the *yvgW* *Bacillus subtilis* chromosomal gene involved in Cd^2+^ ion resistance. FEMS Microbiol Lett 208:105-109.

8. Bruins MR, Kapil S, Oehme FW. 2000. Microbial resistance to metals in the environment. Ecotoxicol Environ Saf 45:198-207.

9. Beveridge TJ, Murray RGE. 1980. Sites of metal deposition in the cell wall of *Bacillus subtilis*. J Bacteriol 141:876-887.

10. Fang Z, Dos Santos PC. 2015. Protective role of bacillithiol in superoxide stress and Fe-S metabolism in *Bacillus subtilis*. Microbiologyopen 4:616-631.

11. Pacheco CC, Passos JOF, Castro AR, Moradas-Ferreira P, Marco PD. 2008. Role of respiration and glutathione in cadmium-induced oxidative stress in *Escherichia coli* K-12. Arch Microbio 189:271-278.

12. Helbig K, Grosse C, Nies DH. 2008. Cadmium toxicity in glutathione mutants of *Escherichia coli*. J Bacteriol 190:5439-5454.

13. Mitra RS, Gray RH, Chin B, Bernstein IA. 1975. Molecular mechanisms of accommodation in *Escherichia coli* to toxic levels of Cd^2+^. J Bacteriol 121:1180-1188.

14. Laddaga RA, Silver S. 1985. Cadmium Uptake in *Escherichia coli* K-12. J Bacteriol 162:1100-1105.

15. Ferianc P, Farewell A, Nystrom T. 1998. The cadmium-stress stimulon of *Escherichia coli* K-12. Microbiology 144 1045-1050.

16. Nies DH. 2003. Efflux-mediated heavy metal resistance in prokaryotes. FEMS Microbiol Rev 27:313-339.

17. Colaço HG, Santo PE, Matias PM, Bandeiras TM, Vicente JB. 2016. Roles of *Escherichia coli* ZinT in cobalt, mercury and cadmium resistance and structural insights into the metal binding mechanism. Metallomics Integrated Biometal Science 8:327-336.

18. Nies DH. 1995. The cobalt, zinc, and cadmium efflux system CzcABC from *Alcaligenes eutrophus* functions as a cation-proton antiporter in *Escherichia coli*. J Bacteriol 10:2707-2712.

19. Li J, Liu Y-R, Zhang L-M, He J-Z. 2019. Sorption mechanism and distribution of cadmium by different microbial species. J ENVIRON MANAGE 237:552-559.

20. Hassan MET, Daniel vdL, Dirk S, Ute R, Nuzha tA, Max M. 1999. Identification of a gene cluster, *czr*, involved in cadmium and zinc resistance in *Pseudomonas aeruginosa*. Gene 238:417-425.

21. Lee SW, Glickmann E, Cooksey DA. 2001. Chromosomal locus for cadmium resistance in *Pseudomonas putida* consisting of a cadmium-transporting ATPase and a MerR family response regulator. APPL ENVIRON MICROB 67:1437-1444.

22. Shamim S, Rehman A, Qazi MH. 2014. Cadmium-resistance mechanism in the bacteria *Cupriavidus metallidurans* CH34 and *Pseudomonas putida* mt2. Arch Environ Contam Toxicol 67:149-157.

23. Manara A, DalCorso G, Baliardini C, Farinati S, Cecconi D, Furini A. 2012. *Pseudomonas putida* response to cadmium: changes in membrane and cytosolic proteomes. J Proteome Res 11:4169-4179.

24. Zheng C, Zhang L, Chen M, Zhao XQ, Duan Y, Meng Y, Zhang X, Shen RF. 2018. Effects of cadmium exposure on expression of glutathione synthetase system genes in *Acidithiobacillus ferrooxidans*. Extremophiles 22:895-902.

25. Ramos-Zuniga J, Gallardo S, Martinez-Bussenius C, Norambuena R, Navarro CA, Paradela A, Jerez CA. 2019. Response of the biomining *Acidithiobacillus ferrooxidans* to high cadmium concentrations. J Proteomics 198:132-144.

26. Zheng C, Chen M, Tao Z, Zhang L, Zhang XF, Wang J-Y, Liu J. 2015. Differential expression of sulfur assimilation pathway genes in *Acidithiobacillus ferrooxidans* under Cd^2+^ stress: evidence from transcriptional, enzymatic, and metabolic profiles. Extremophiles 19:429-436.

27. Bhanoori M, Venkateswerlu G. 2000. In vivo chitin-cadmium complexation in cell wall of *Neurospora crassa*. Biochim Biophys Acta 1523:21-28.

28. Kapoor M, Sreenivasan GM, Goel N, Lewis J. 1990. Development of thermotolerance in *Neurospora crassa* by heat shock and other stresses eliciting peroxidase induction. J Bacteriol 172:2798-2801.

29. Kiranmayi P, Tiwari A, Sagar KP, Haritha A, Maruthi Mohan P. 2009. Functional characterization of *tzn1* and *tzn2*-zinc transporter genes in *Neurospora crassa*. Biometals 22:411-420.

30. Vido K, Spector D, Lagniel G, Lopez S, Toledano MB, Labarre J. 2001. A proteome analysis of the cadmium response in *Saccharomyces cerevisiae*. J Biol Chem 276:8469-8474.

31. Momose Y, Iwahashi H. 2001. Bioassay of cadmium using a DNA microarray: Genome-wide expression patterns of *Saccharomyces cerevisiae* response to cadmium. Environ Toxicol Chem 20:2353-2360.

32. Xiong B, Zhang L, Xu H, Yang Y, Jiang L. 2015. Cadmium induces the activation of cell wall integrity pathway in budding yeast. Chem Biol Interact 240:316-323.

33. Liu XF, Supek F, Nelson N, Culotta VC. 1997. Negative control of heavy metal uptake by the *Saccharomyces cerevisiae* BSD2 gene. J Biol Chem 272:11763-11769.

34. Liu XF, Culotta VC. 1999. Post-translation control of Nramp metal transport in yeast. Role of metal ions and the BSD2 gene. J Biol Chem 274:4863-4868.

35. Mendoza-Cozatl D, Loza-Tavera H, Hernandez-Navarro A, Moreno-Sanchez R. 2005. Sulfur assimilation and glutathione metabolism under cadmium stress in yeast, protists and plants. FEMS Microbiol Rev 29:653-671.

36. Gitan RS, Luo H, Rodgers J, Broderius M, Eide D. 1998. Zinc-induced Inactivation of the Yeast ZRT1 Zinc Transporter Occurs through Endocytosis and Vacuolar Degradation. J BIOL CHEM 273:28617-28624.

37. Shiraishi E, Inouhe M, Joho M, Tohoyama H. 2000. The cadmium-resistant gene,CAD2, which is a mutated putative copper-transporter gene (PCA1), controls the intracellular cadmium-level in the yeast *S. cerevisiae*. Curr Genet 37:79-86.

38. Kern AL, Bonatto D, Dias JF, Yoneama M-L, Brendel M, Henriques JAP. 2005. The function of Alr1p of *Saccharomyces cerevisiae* in cadmium detoxification: Insights from phylogenetic studies and particle-induced X-ray emission. Biometals 18:31-41.

39. Cagnac O, Bourbouloux A, Chakrabarty D, Zhang M-Y, Delrot S. 2004. AtOPT6 Transports Glutathione Derivatives and Is Induced by Primisulfuron. Plant Physiol 135:1378-1387.

40. Adle DJ, Sinani D, Kim H, Lee J. 2007. A Cadmium-transporting P 1B -type ATPase in Yeast *Saccharomyces cerevisia*. J Biol Chem 282:947-955.

41. Li ZS, Lu YP, Zhen RG, Szczypka M, ., Thiele DJ, Rea PA. 1997. A new pathway for vacuolar cadmium sequestration in *Saccharomyces cerevisiae*: YCF1-catalyzed transport of bis(glutathionato)cadmium. Proc Natl Acad Sci U S A 94:42-47.

42. Sharma KG, Mason DL, Liu G, Rea PA, Bachhawat AK, Michaelis S. 2002. Localization, regulation, and substrate transport properties of Bpt1p, a *Saccharomyces cerevisiae* MRP-type ABC transporter. Eukaryot Cell 1:391-400.

43. Fauchon M, Lagniel G, Aude JC, Lombardia L, Soularue P, Petat C, Marguerie G, Sentenac A, Werner M, Labarre J. 2002. Sulfur sparing in the yeast proteome in response to sulfur demand. Mol Cell 9:713-723.

44. Harumi Oshiquiri L, Roterdanny Araujo Dos Santos K, Alves Ferreira Junior S, Stecca Steindorff A, Rodrigues Barbosa Filho J, Marcolino Mota T, Jose Ulhoa C, Castro Georg R. 2019. *Trichoderma harzianum* transcriptome in response to cadmium exposure. Fungal Genet Biol 134:103281.

45. Andrea G. 2003. Growth inhibition of the filamentous fungus *Aspergillus nidulans* by cadmium: an antioxidant enzyme approach. J Gen Appl Microbiol 49:63–74.

46. Balaska S, Myrianthopoulos V, Tselika M, Hatzinikolaou DG, Mikros E, Diallinas G. 2017. NmeA, a novel efflux transporter specific for nucleobases and nucleosides, contributes to metal resistance in *Aspergillus nidulans*. Mol Microbiol 105:426-439.

47. Chakraborty S, Mukherjee A, Khuda-Bukhsh AR, Das TK. 2014. Cadmium-induced oxidative stress tolerance in cadmium resistant *Aspergillus foetidus*: its possible role in cadmium bioremediation. Ecotoxicol Environ Saf 106:46-53.

48. Baik WY, Bae JH, Cho KM, Hartmeier W. 2002. Biosorption of heavy metals using whole mold mycelia and parts thereof. Bioresource Technology 81:167-170.

49. Chen M, Wang L, Hou J, Yang S, Zheng X. 2018. Mycoextraction: Rapid Cadmium Removal by Macrofungi-Based Technology from Alkaline Soil. Minerals 8:589-605.

50. Li X, Wang Y, Pan Y, Yu H, Zhang X, Shen Y, Jiao S, Wu K, La G, Yuan Y, Zhang S. 2017. Mechanisms of Cd and Cr removal and tolerance by macrofungus *Pleurotus ostreatus* HAU-2. J Hazard Mater 330:1-8.

51. Chuang HW, Wang IW, Lin SY, Chang YL. 2009. Transcriptome analysis of cadmium response in *Ganoderma lucidum*. FEMS Microbiol Lett 293:205-213.

52. Yu Z, Zhang T, Zhu Y. 2020. Whole-genome re-sequencing and transcriptome reveal cadmium tolerance related genes and pathways in *Chlamydomonas reinhardtii*. Ecotoxicol Environ Saf 191:110231-110239.

53. Macfie SM, Welbourn PM. 2000. The Cell Wall as a Barrier to Uptake of Metal Ions in the Unicellular Green Alga *Chlamydomonas reinhardtii* (Chlorophyceae). Arch Environ Contam Toxicol 39:413-419.

54. Siripornadulsil S, Traina S, Verma DP, Sayre RT. 2002. Molecular mechanisms of proline-mediated tolerance to toxic heavy metals in transgenic microalgae. Plant Cell 14:2837-2847.

55. Carr HP, Carino FA, Yang MS, Wong MH. 1998. Characterization of the cadmium-binding capacity of *Chlorella vulgaris*. Bull Environ Contam Toxicol 60:433-440.

56. Cheng J, Qiu H, Chang Z, Jiang Z, Yin W. 2016. The effect of cadmium on the growth and antioxidant response for freshwater algae *Chlorella vulgaris*. Springerplus 5:1290.

57. Ting YP, Lawson F, Prince IG. 1989. Uptake of cadmium and zinc by the alga *Chlorella vulgaris*: part 1. Individual ion species. Biotechnol Bioeng 34:990-999.

58. Mills RF, Krijger GC, Baccarini PJ, Hall JL, Williams LE. 2003. Functional expression of AtHMA4, a P1B-type ATPase of the Zn/Co/Cd/Pb subclass. Plant J 35:164-176.

59. Gravot A, Lieutaud A, Verret F, Auroy P, Vavasseur A, Richaud P. 2004. AtHMA3, a plant P1B-ATPase, functions as a Cd/Pb transporter in yeast. FEBS Lett 561:22-28.

60. Adle DJ, Sinani D, Kim H, Lee J. 2007. A Cadmium-transporting P 1B -type ATPase in Yeast Saccharomyces cerevisiae. J Biol Chem 282: 947-955.

61. Sébastien T. 2003. AtNRAMP3, a multispecific vacuolar metal transporter involved in plant responses to iron deficiency. Plant J 5: 685-695.

62. Bovet L, Eggmann T, MEYLAN EM, Polier JE, Martinoia E. 2003. Transcript levels of AtMRPs after cadmium treatment: Induction of AtMRP3. Plant Cell Environ 26:371-381.

63. Guerinot ML. 2000. The ZIP family of metal transporters. Biochimica et Biophysica Acta (BBA) - Biomembranes 1465:190-198.

64. Brembu T, Jorstad M, Winge P, Valle KC, Bones AM. 2011. Genome-wide profiling of responses to cadmium in the diatom *Phaeodactylum tricornutum*. ENVIRON SCI TECHNOL 45:7640-7647.

65. Jia H, Wang X, Dou Y, Liu D, Si W, Fang H, Zhao C, Chen S, Xi J, Li J. 2016. Hydrogen sulfide - cysteine cycle system enhances cadmium tolerance through alleviating cadmium-induced oxidative stress and ion toxicity in *Arabidopsis* roots. Sci Rep 6:39702-39715.

66. Dominguez-Solis JR, Gutierrez-Alcala G, Romero LC, Gotor C. 2001. The Cytosolic O-Acetylserine(thiol)lyase Gene Is Regulated by Heavy Metals and Can Function in Cadmium Tolerance. J BIOL CHEM 276:9297-9302.

67. Emiko Harada, Yube Yamaguchi, Nozomu Koizumi, Sano Hiroshi. 2002. Cadmium stress induces production of thiol compounds and transcripts for enzymes involved in sulfur assimilation pathways in *Arabidopsis*. J Plant Physiol 159:445-448.

68. Zhu XF, Wang ZW, Dong F, Lei GJ, Shi YZ, Li GX, Zheng SJ. 2013. Exogenous auxin alleviates cadmium toxicity in *Arabidopsis thaliana* by stimulating synthesis of hemicellulose 1 and increasing the cadmium fixation capacity of root cell walls. J Hazard Mater 263 398-403.

69. Yamaguchi C, Takimoto Y, Ohkama-Ohtsu N, Hokura A, Shinano T, Nakamura T, Suyama A, Maruyama-Nakashita A. 2016. Effects of Cadmium Treatment on the Uptake and Translocation of Sulfate in *Arabidopsis thaliana*. Plant Cell Physiol 57:2353-2366.

70. Nourimand M, Todd CD. 2016. Allantoin Increases Cadmium Tolerance in *Arabidopsis* via Activation of Antioxidant Mechanisms. Plant Cell Physiol 57:2485-2496.

71. VAN DE MORTEL JE, SCHAT H, MOERLAND PD, VAN THEMAAT EVL, VAN DER ENT S, BLANKESTIJN H, GHANDILYAN A, TSIATSIANI S, AARTS MGM. 2008. Expression differences for genes involved in lignin, glutathione and sulphate metabolism in response to cadmium in *Arabidopsis thaliana* and the related Zn/Cd-hyperaccumulator *Thlaspi caerulescens*. Plant Cell Environ 31:301-324.

72. Boominathan R, Doran PM. 2003. Organic acid complexation, heavy metal distribution and the effect of ATPase inhibition in hairy roots of hyperaccumulator plant species. J Biotechnol 101:131-146.

73. Pence NS, Larsen PB, Ebbs SD, Letham DLD, Lasat MM, Garvin DF, Eide D, Kochian LV. 2000. The molecular physiology of heavy metal transport in the Zn/Cd hyperaccumulator *Thlaspi caerulescens*. Proc Natl Acad Sci U S A 97:4956-4960.

74. Bernard C, Roosens N, Czernic P, Lebrun M, Verbruggen N. 2004. A novel CPx-ATPase from the cadmium hyperaccumulator *Thlaspi caerulescens*. FEBS Lett 569:140-148.

75. Persans MW, Nieman K, Salt DE. 2001. Functional activity and role of cation-efflux family members in Ni hyperaccumulation in *Thlaspi goesingense*. Proc Natl Acad Sci U S A 98:9995-10000.

76. Hu YT, Ming F, Chen WW, Yan JY, Xu ZY, Li GX, Xu CY, Yang JL, Zheng SJ. 2012. TcOPT3, a Member of Oligopeptide Transporters from the Hyperaccumulator *Thlaspi caerulescens*, Is a Novel Fe/Zn/Cd/Cu Transporter. PLoS One 7:e38535.

77. Benzarti S, Hamdi H, Mohri S, Ono Y. 2010. Response of antioxidative enzymes and apoplastic bypass transport in *Thlaspi caerulescens* and R*aphanus sativus* to cadmium stress. Int J Phytoremediation 12:733-744.

78. Mostofa MG, Rahman A, Ansary MM, Watanabe A, Fujita M, Tran LS. 2015. Hydrogen sulfide modulates cadmium-induced physiological and biochemical responses to alleviate cadmium toxicity in rice. Sci Rep 5:14078.

79. Zhu CQ, Cao XC, Zhu LF, Hu WJ, Hu AY, Bai ZG, Zhong C, Sun LM, Liang QD, Huang J, Yang SX, Zhang JH, Jin QY. 2018. Ammonium mitigates Cd toxicity in rice (*Oryza sativa*) via putrescine-dependent alterations of cell wall composition. Plant Physiol Biochem 132:189-201.

80. Cao F, Chen F, Sun H, Zhang G, Chen ZH, Wu F. 2014. Genome-wide transcriptome and functional analysis of two contrasting genotypes reveals key genes for cadmium tolerance in barley. BMC Genomics 15:611.
