# Supplementary material for: Cross-Kingdom Comparative Transcriptomics Reveals Conserved Genetic Modules in Response to Cadmium Stress
Source: mSystems. 2021 Dec 7;6(6):e01189-21. doi: 10.1128/mSystems.01189-21 (PMC8651089; doi:10.1128/mSystems.01189-21)
Supplement: TABLE S3 [file msystems.01189-21-st003.docx]

| **Gene** | **Primer (5’-3’)** | **Ct value (mean±SEM) in control** | **Ct value (mean±SEM) in Cd treatment** | **Melt Temp in control (℃)** | **Melt Temp in Cd treatment (℃)** |
| --- | --- | --- | --- | --- | --- |
| B21_RS09935 | Forward: ACCGAAAGCTGAGTGACTGG  Reserve: ATGCCGTCCTCAATGCCAAT | 25.85±0.13 | 18.90±0.11 | 81.00 | 81.00 |
| B21_RS02785 | Forward: GGCGACACCCAGAATACCAA  Reserve: TACGGGTGTGTTCGTACTGC | 28.31±0.09 | 20.39±0.13 | 85.00 | 85.50 |
| B21_RS17315 | Forward: GGGCGTTAGCGCTTTAATGG  Reserve: GCGGCGACTTCAATCACATC | 29.29±0.14 | 23.52±0.19 | 86.50 | 86.50 |
| B21_RS17320 | Forward: TGATGGTGCGCAAAACTGTG  Reserve: AGTCCATCCGTCTCTTTCGC | 25.89±0.06 | 21.06±0.05 | 84.00 | 84.00 |
| B21_RS13605 | Forward: CCATCGAACACGGCAACAAG  Reserve: TAAACGTCTCAACCCCCACG | 28.47±0.13 | 23.32±0.09 | 87.00 | 87.00 |
| B21_RS05900 | Forward: TCGAAGTTCAGTCAACGCCA  Reserve: CGGAAAGATTTTGCGCCCAT | 27.33±0.05 | 22.04±0.07 | 84.00 | 84.00 |
| B21_RS22395 | Forward: TCGCAACCAACCGATGATGA  Reserve: CATGGAAATGTTCCGGCGAC | 28.03±0.07 | 23.59±0.15 | 83.50 | 84.00 |
| B21_RS13600 | Forward: TTCGGCCGGATATTCCAGTG  Reserve: TGTTCCCACAGTTTGCCGTA | 28.13±0.12 | 23.37±0.04 | 84.00 | 84.50 |
| B21_RS13610 | Forward: CTGCCAGTCCGGGAAAGATT  Reserve: TTTAAGCGCATCAACCACGC | 28.51±0.40 | 23.11±0.12 | 88.00 | 88.00 |
| B21_RS19915 | Forward: GGCGATAACTGGTATCCGGG  Reserve: AAGGCGATGCATTGTCTCCA | 22.44±0.10 | 18.16±0.05 | 84.00 | 84.00 |
| B21_RS20540 | Forward: GTGGCTGTGGAACTCGGTAA  Reserve: CATTCCTTTCAGCAGCGTCG | 23.61±0.06 | 27.9±0.14 | 87.00 | 86.50 |
| B21_RS20565 | Forward: AAGCGCTTCTACCAACGTCA  Reserve: GGTTGCTGCCAGAGAGAGTT | 17.94±0.06 | 24.05±0.44 | 82.00 | 82.00 |
| B21_RS21615 | Forward: CTCGTATCAAACGCCAGGGT  Reserve: CCTGTCCACACCCAGATGTC | 20.91±0.07 | 23.93±0.06 | 85.00 | 85.00 |
| B21_RS07750 | Forward: TGAGGCAATTGGCGTTGTTG  Reserve: GCTCCGGTGCAGGATTATCA | 20.6±0.25 | 24.25±0.25 | 82.00 | 82.00 |
| B21_RS21625 | Forward: AGTCTGTGAATTCTGGGCCG  Reserve: TGCAAAAACTCGTGTGCTCG | 18.68±0.05 | 21.12±0.21 | 87.00 | 87.00 |
| ihfB | Forward: GCCAAGACGGTTGAAGATGC  Reserve: GTCTTCGGATTACGTCCGGT | 24.49±0.03 | 24.28±0.11 | 85.00 | 85.00 |
| YLR303W | Forward: CCCGTTTCCAAAACCCAACC  Reserve: AGTGTGTGCCAAACCTTGGA | 28.85±0.15 | 26.02±0.31 | 83.50 | 83.50 |
| YIR017C | Forward: ACATTGCAAGCAGGAAGGGT  Reserve: CGCGGAGCTTCTTAAACTGC | 29.23±0.08 | 27.45±0.69 | 83.50 | 83.50 |
| YFL057C | Forward: GCGCCTCTGAACAAACAGATG  Reserve: TCCTTCCTCCAACCAATGGAAA | 24.64±0.07 | 23.92±0.60 | 81.00 | 81.00 |
| YER091C | Forward: GAAGCTGCCGGTATCAAGGT  Reserve: GGCAGCCCAGGTGTAGTAAG | 26.38±0.07 | 24.58±0.33 | 80.00 | 80.00 |
| YKL001C | Forward: AGAAGTCGCTGAGCAAAGGG  Reserve: TTCAACCGTCTTCTGGTCGG | 27.57±0.21 | 26.24±0.39 | 81.50 | 81.50 |
| YLL061W | Forward: GTGGGTAGTCCCAGTTCTGC  Reserve: TGTCTCCAGTCTCTGCGGTA | 24.41±0.10 | 23.49±0.29 | 77.50 | 77.50 |
| YDR502C | Forward: TCCACCGCTGACTTGAGAAC  Reserve: CCTTGAGGACCACCGATGAC | 25.65±0.14 | 24.63±0.40 | 79.00 | 79.00 |
| YLR180W | Forward: CTTGGACGCTTGTTTAGCCG  Reserve: ATAGTCGAAACCCTTGGCGG | 24.34±0.34 | 23.26±0.48 | 82.50 | 82.50 |
| YPL274W | Forward: CCTTGTTGCGGTTGTGTCAG  Reserve: GTCCTCCTTTTTCGAGGCCA | 26.23±0.60 | 26.13±0.69 | 82.00 | 82.00 |
| YLL060C | Forward: TGAACAAACGCGCAGAGTTG  Reserve: GCGAAGTCCCCACTCTTTGT | 25.21±0.40 | 25.18±0.60 | 82.00 | 82.00 |
| YGR055W | Forward: CAACGGTGCCTCCGATTTTG  Reserve: GAGGAAACGGCGAAAACACC | 26.25±0.35 | 24.81±0.50 | 81.50 | 81.50 |
| YFR030W | Forward: TACGACAATGCTACCGGCTC  Reserve: AGGCGACACTTTGTACCTCG | 25.87±0.11 | 25.56±0.37 | 82.50 | 82.50 |
| YPR167C | Forward: ATTGGACGTTCGAGCAGGTT  Reserve: TCACCTTCCTTGACGGGTTG | 25.85±0.20 | 26.50±0.67 | 79.00 | 79.00 |
| YGL255W | Forward: ATGGACCCTGCTTATGGTGC  Reserve: TCGTGGGTATGGTCATGGGA | 25.97±0.52 | 28.52±0.58 | 81.50 | 81.50 |
| ALG9 | Forward: CACGGATAGTGGCTTTGGTGAACAATTAC  Reserve: TATGATTATCTGGCAGCAGGAAAGAACTTGGG | 25.65±0.10 | 26.78±0.70 | 81.50 | 81.50 |
| CHLRE_07g321800v5 | Forward: CGGACGACAACAAGCCAATC  Reserve: GCCGGTCGCGTTATAGAAGA | 25.59±0.20 | 20.07±0.27 | 86.00 | 86.00 |
| CHLRE_03g180750v5 | Forward: TTTCAAGCACCTCTCGGGTC  Reserve: TCGGGCACCAGGTAGTGATA | 29.88±0.18 | 25.10±0.05 | 84.50 | 84.50 |
| CHLRE_01g031500v5 | Forward: TGTGTACTACGAGGGCCTGA  Reserve: GAAGGAGTTGGCAGGGTTGT | 27.38±0.11 | 24.37±0.11 | 89.00 | 89.00 |
| CHLRE_03g204250v5 | Forward: TCAATGGCCGCGAGTACAAG  Reserve: CATGTGCAGAGAGCCGGTAA | 25.98±0.20 | 22.00±0.10 | 89.00 | 89.00 |
| CHLRE_06g250200v5 | Forward: ACTGGCATGGTGATGGTGTT  Reserve: TCCGAGGTGAAGCCAATCTC | 24.38±0.06 | 21.58±0.12 | 83.50 | 84.00 |
| CHLRE_01g029000v5 | Forward: CCCGAGTACGGCATCTTTCT  Reserve: GCGTGTCGTCAAACTCATCC | 27.05±0.17 | 24.61±0.27 | 89.50 | 89.50 |
| CHLRE_07g351950v5 | Forward: GACACCTACGATGCCGAGAG  Reserve: TCCGATATCACCATTCCGGC | 25.23±0.12 | 22.76±0.12 | 85.50 | 85.50 |
| CHLRE_14g630871v5 | Forward: ACCACCAGCACGACTCCT  Reserve: GGACCCCCTGGAACACATAC | 25.75±0.12 | 23.40±0.04 | 90.50 | 90.50 |
| CHLRE_12g525650v5 | Forward: GGATGACGCACCATCGTACA  Reserve: GTCCAGGTAGACCAGGGGAT | 33.97±0.50 | 33.21±0.06 | 82.50 | 82.00 |
| CHLRE_02g145100v5 | Forward: ATGCAGACGCCCATCAACTA  Reserve: CTGAGCCAGTTGGAAGAGGT | 24.88±0.18 | 27.66±0.15 | 88.50 | 88.50 |
| CHLRE_05g248300v5 | Forward: CAGTCCTCCACCATGACAGG  Reserve: TTGAGGCCCTGGTTCAACTG | 26.85±0.16 | 28.58±0.52 | 90.50 | 90.50 |
| CHLRE_12g530900v5 | Forward: ACAGCAGCCAAATCCCTGTT  Reserve: TGGCTCCAGTGACATCGTTC | 22.04±0.21 | 24.67±0.04 | 88.50 | 88.50 |
| CHLRE_12g530400v5 | Forward: CATTACCTCGGGCATCACCA  Reserve: CCACATGACCGCCTTGAGT | 20.25±0.03 | 23.09±0.13 | 86.50 | 86.50 |
| CHLRE_09g396600v5 | Forward: TGGACCACGTAATCACCACC  Reserve: GGGGCTCCTTGGTCACTATC | 24.08±0.24 | 27.23±0.18 | 91.50 | 91.50 |
| a-tubulin | Forward: CTCGCTTCGCTTTGACGGTG  Reserve: CGTGGTACGCCTTCTCGGC | 20.58±0.12 | 20.88±0.10 | 86.00 | 86.00 |

Notes: Ct stands for cycle threshold (Ct) values of each gene. SEM is the Standard error. ihfB, ALG9 and a-tubulin are the reference genes for qPCR of *Escherichia coli*, *Saccharomyces cerevisiae* and *Chlamydomonas reinhardtii*, respectively.
