# Supplementary material for: Cross-Kingdom Comparative Transcriptomics Reveals Conserved Genetic Modules in Response to Cadmium Stress
Source: mSystems. 2021 Dec 7;6(6):e01189-21. doi: 10.1128/mSystems.01189-21 (PMC8651089; doi:10.1128/mSystems.01189-21)
Supplement: TABLE S4 [file msystems.01189-21-st004.docx]

| **Candidated gene** | **Function** | **Full-length CDS primer** | **Restrictive primer** |
| --- | --- | --- | --- |
| B21_RS13195 | Glycine betaine/L-proline ABC transporter | Forward: 5’-TCGACATAGACAAATAAAGGAA-3’  Reserve: 5’-CTGGAAACCGTTGAGGAT-3’ | Forward: 5’-CGGGATCCATGGCAATTAAATTAG-3’  Reserve: 5’-CTAGCTAGCTCAGCCATTATTTACC-3’ |
| YDR135C | ABC type transporter YCF1 | Forward: 5’- ACTACCGTAAAGAACAAGA-3’  Forward: 5’-  TGACCAAACCAGCCTCCA-3’ | Forward: 5’-CGGGATCCATGGCTGGTAATCTTGTTTC-3’  Forward: 5’-CGAGCTCTTAATTTTCATTGACC-3’ |
| CHLRE_13g604150v5 | ABC transporter | Forward: 5’-AGCCATAGCTTTAGCGATGAC-3’  Reserve: 5’-  CGACGCAGAACGCCAATA-3’ | Forward: 5’-CGGAATTCATGGCAACGGGGCCAC-3’  Forward: 5’-ACGCGTCGACTTACGCCGCATTCACT-3’ |
